# Variation in sugarcane biomass composition and enzymatic saccharification of leaves, internodes and roots

**DOI:** 10.1186/s13068-020-01837-2

**Published:** 2020-12-09

**Authors:** Patrick J. Mason, Agnelo Furtado, Annelie Marquardt, Katrina Hodgson-Kratky, Nam V. Hoang, Frederik C. Botha, Gabriella Papa, Jenny C. Mortimer, Blake Simmons, Robert J. Henry

**Affiliations:** 1 Queensland Alliance for Agriculture and Food Innovation (QAAFI), Level 2, Queensland Biosciences Precinct [#80], The University of Queensland, St Lucia, QLD 4072 Australia; 2 Commonwealth Scientific and Industrial Research Organisation (CSIRO), Level 3, Queensland Biosciences Precinct [#80], The University of Queensland, St Lucia, QLD 4072 Australia; 3 Sugar Research Australia Limited (SRA), PO Box 86, Indooroopilly, QLD 4068 Australia; 4 College of Natural Science, Seoul National University, Seoul, 08826 Republic of Korea; 5 Amyris, 5885 Hollis St, Ste. 100, Emeryville, CA 94608 USA; 6 Lawrence Berkeley National Laboratory (LBNL), Joint Bioenergy Institute (JBEI), 5885 Hollis St, Emeryville, CA 94608 USA

**Keywords:** Carbon partitioning, Sugarcane *(saccharum* spp. hybrids), Compositional analysis, Cell wall, Soluble sugars

## Abstract

**Background:**

The composition of biomass determines its suitability for different applications within a biorefinery system. The proportion of the major biomass fractions (sugar, cellulose, hemicellulose and lignin) may vary in different sugarcane genotypes and growth environments and different parts of the plant. This study investigated the composition of mature and immature internodes, roots and mature leaves of sugarcane.

**Results:**

Internodes were found to have a significantly larger alcohol-soluble component than leaves and roots. The primary difference between the immature and mature internodes was the ratio of soluble sugars. In mature tissues, sucrose content was significantly higher, whereas in immature internodal tissues there was lower sucrose and heightened concentrations of reducing sugars. Carbon (C) partitioning in leaf tissues was characterised by low levels of soluble components and high “other” and cell wall fractions. Root tissue had low ratios of soluble fractions relative to their cell wall contents, indicating a lack of storage of soluble carbon. There was no significant difference in the ratio of the major cell wall fractions between the major organ types. Characterisation of individual non-cellulosic monomers indicated leaf and root tissues had significantly higher arabinose and galactose fractions. Significantly larger proportions of syringyl lignin compounds and the hydroxycinnamic compound, *p-*coumaric acid were observed in mature internodal tissues compared to the other tissue types. Tissue-specific differences in composition were shown to greatly affect the recalcitrance of the cell wall to enzymatic saccharification.

**Conclusions:**

Overall, this study displayed clear evidence of the differential partitioning of C throughout the sugarcane plant in specific organs. These organ-specific differences have major implications in their utility as a bioproduct feedstock. For example, the inclusion of trash (leaves) with the culms (internodes) may alter processing efficiency.

## Background

Sugarcane (*Saccharum* spp. hybrids), of the *Saccharum* genus, is a crop plant utilised primarily in the production of sucrose. More recently, the need has arisen to diversify the sugarcane industry beyond the focused production of sucrose, due to the falling prices of raw sugar throughout the past decade [1]. In response to this, the insoluble (fibre) fraction of sugarcane has been targeted as an ideal feedstock for the production of energy products, polymers and non-fossil-based chemicals [2], within a biorefinery system [3–8]. Producing a large amount of sugarcane fibre high in cellulose and low in hemicellulose and lignin would be highly desirable for the biorefinery industry [9]. Further, producing a hemicellulose fraction with low levels of pentose molecules and lignin would also be of significant value [10, 11]. The aforementioned factors are intrinsically linked to the way carbon (C) is partitioned within the mature sugarcane plant. Understanding where this C is partitioned within mature sugarcane plants will be key to the future development of this crop for use in biorefinery systems [12].

Internodes, leaves, and roots utilise C in different ways throughout their life cycle, which in turn leads to distinct compositional profiles. Leaves are a source tissue, exporting over 80% of fixed photosynthates to sink tissues at maturity [13]. Conversely, root and internodal tissue are net importers of C in the form of sucrose, i.e. sink tissues, which is cleaved into uridine diphosphate glucose (UDP-glucose) to drive cell wall expansion, hydrolysed into glucose driving respiration, or retained as sucrose for storage, [14, 15]. There is an additional degree of difference in C partitioning between root and internodes. C partitioning in sugarcane internodal tissue is unique from other organs, as C utilisation changes from one focused primarily on respiration, cell wall and protein deposition i.e. meristematic sink, to that of storage of simple sugars in the form of sucrose, i.e. a storage sink [16]. Compositionally, the photosynthetic nature of sugarcane leaves leads to a heightened protein profile in comparison to mature internodal tissues [17, 18]. Further, cell wall analysis of mature sugarcane leaves displayed significantly higher lignin and arabinose profiles in comparison to whole culm samples [19]. A comparison of saccharification efficiency of the whole leaf vs. the whole culm displayed significant differences between the two tissue types [20]. There is a dearth of studies in sugarcane root composition [21], therefore no comparisons in composition or saccharification efficiency have been made.

Internodal tissue is well characterised in sugarcane, whereby C partitioning into competing pools has been found to change greatly throughout maturity [13, 22–24]. Throughout internodal maturation, the bulk of C is allocated to the cell wall and respiratory pools in immature internodes, whereas in mature internodes the bulk of C is allocated to sucrose storage [16]. From a compositional perspective, C partitioning has a direct effect on the ratio of insoluble (cell wall, protein and other secondary metabolites) and soluble components (sugars). C partitioning to the major components in the insoluble fraction has been shown to fluctuate greatly during maturation, as displayed by high protein and hemicellulose in very young internodes, followed by a gradual increase in cellulose and lignin content until tissue maturity, between internodes 5 and 8 [25]. Within the soluble fraction, which is dominated by hexose and sucrose, hexose content is significantly higher in immature internodes, whereas in mature internodes sucrose dominates [23, 24, 26–29].

Due to the economic importance of the sugarcane culm, the composition of individual internodes has been well characterised between varying levels of maturity, and between genotypes [16, 25, 30–33]. The composition in the other important organs of commercial sugarcane, i.e. roots and leaves, has not been characterised to the same extent as internodal tissues. This limits our understanding of compositional differences between different parts of the sugarcane plant, which in terms of total biomass produced are proportional to the culm [34]. Differences in composition between the major organs of sugarcane may have implications regarding their use in specific biorefinery applications. In this study, a combination of methods was utilised to evaluate the ratio of insoluble and soluble components of six different tissues, encapsulating the major organs of two commercial sugarcane genotypes. Characterising organ-specific compositional differences within sugarcane may have utility in the inclusion of by-products such as “cane trash” in biomass processing.

## Results

**Fig. 1:**
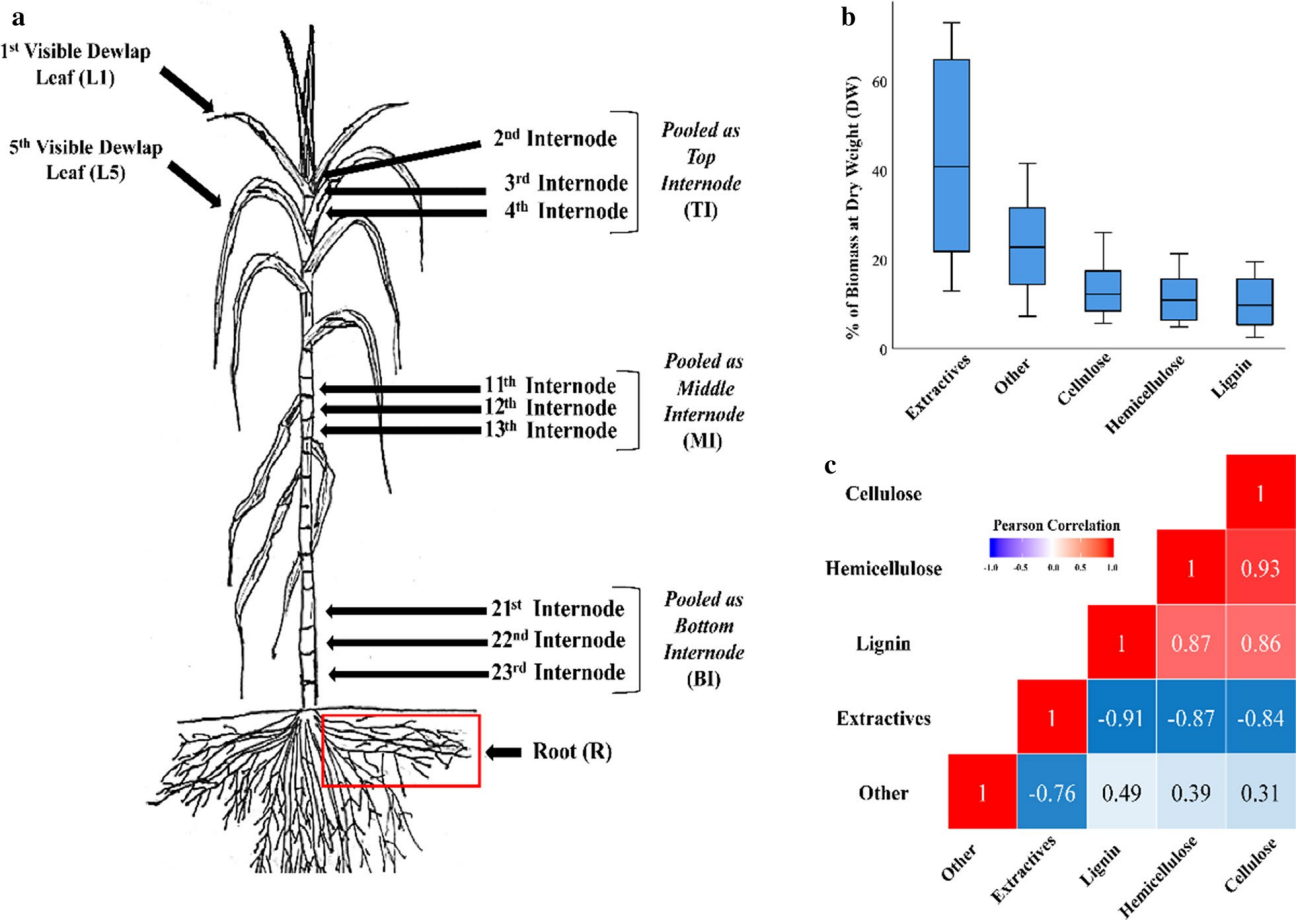
Sugarcane sampling schematic and overview of compositional statistics. **a** Labelled sugarcane plant schematic, as utilised in this study. Internodes and leaves labelled in line with McCormick et al. [43]. **b,** **c** Combined leaf, root and internode data for hemicellulose, cellulose, lignin, other and total extractives (*N* = 36). **a** Box plot of cellulose, hemicellulose, lignin, other and extractives. **b** Pearson correlation analysis between determined compositions, cellulose, hemicellulose, lignin, other and extractives. Bolder colours indicate a stronger correlation. The red colour describes a positive correlation, whilst the blue describes a negative correlation. All values reported and compared at DW

This study included the analysis of two commonly utilised commercial sugarcane genotypes KQ228 and Q208, within the Australian sugar industry. No significant compositional differences were observed between the two genotypes, as was expected due to their common lineage and phenotype [35]. Summary results for total biomass composition analysis on all 36 sugarcane samples utilised in this study are presented in Table 1. The percentage extractives on a DW basis fell between 12.9 and 73%. On an extractive free basis, or % of alcohol insoluble residue (AIR), the results were as follows, 14.5–31.3% cellulose, 9.9–25.5% hemicellulose, 7.9–32.2% lignin, 13.4–66% other and 0–6.2% ash. Figure 1a (values presented as % at DW) displays the same data as presented in Table 1, with cellulose, hemicellulose, lignin and other presented as % in AIR, whilst total extractives were presented as a % at DW. Within the five major components (other, extractives, lignin, hemicellulose and cellulose), total extractives have the widest variation compared to the other four ranges. A Pearson correlation test indicated a similar positive correlation between the major cell wall components cellulose, hemicellulose and lignin (Fig. 1b). Cellulose has a similar correlation with lignin (*R*^2^ = 0.86, *p* < 0.001), as hemicellulose and lignin (*R*^*2*^ = 0.87, *p* < 0.001), whilst cellulose and hemicellulose have a higher correlation (*R*^2^ = 0.93, *p* < 0.001). Total extractives were negatively correlated with lignin (*R*^2^ = − 0.91, *p* < 0.001), hemicellulose (*R*^2^ = − 0.87, *p* < 0.001) and cellulose (*R*^2^ = − 0.84, *p* < 0.001) contents. The other fraction had a high negative correlation with extractives (*R*^2^ = − 0.76, *p* < 0.001) and a weaker positive correlation with lignin (*R*^*2*^ = 0.49, *p* < 0.001), hemicellulose (*R*^*2*^ = 0.39, *p* < 0.001) and cellulose (*R*^*2*^ = 0.31, *p* < 0.001).

**Table 1:** Compositional summary statistics for 36 sugarcane samples across two commercial genotypes

	% Cellulose (AIR)	% Hemicellulose (AIR)	% Lignin (AIR)	% Ash (AIR)	% Other (AIR)	% Total extractives (DW)
Range	16.7	15.6	24.3	6.2	52.7	60.1
Minimum	14.5	9.9	7.9	0	13.4	12.9
Maximum	31.3	25.5	32.2	6.2	66	73
S.D	5.3	4.4	4.9	1.6	11.4	21.9
Mean	23.2	19	17.5	1.3	40.3	42.1
Literature Range	33–60	21–34	4–31	–	–	5–68

All values are reported on an extractive free basis, otherwise known as alcohol-insoluble residue (AIR), except for total extractives, which are reported on a dry weight (DW) basis. Literature range adapted from [19, 30, 36]. It is important to note here that the reason cellulose and hemicellulose values fall outside of the literature range is likely due to the inclusion of the large “other” fraction included in the AIR calculation. Further, the mixed-link β-glucan (MLG) fraction was also subtracted from the cellulose fraction, which was not the case in literature range studies

### Total biomass comparison

**Fig. 2:**
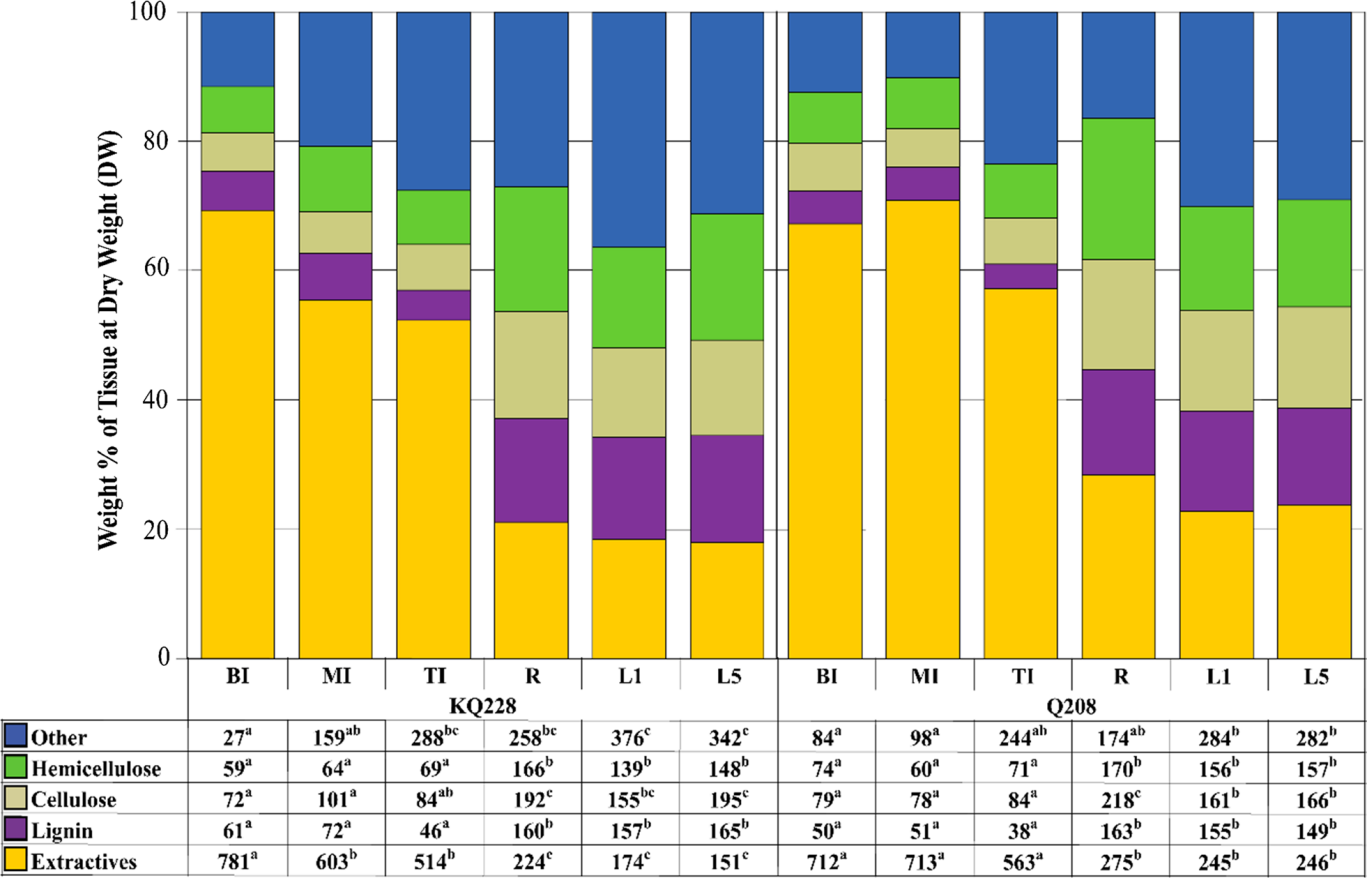
Contribution of *Saccharum* spp. hybrid major tissue components to total tissue composition. The consolidation of total tissue weight percentages of other (dark blue), hemicellulose (green), cellulose (light brown), lignin (purple), and extractives (yellow) to equal 100% is given for each tissue. Numbers in table are in the units of mg g^−1^ total tissue at DW. Extractives were calculated as the loss of mass from AIR after alcohol extraction. KQ228 and Q208 refer to the *Saccharum* spp. hybrid genotype. Abbreviations, TI: top internode; MI: middle internode; BI: bottom internode; first visible dewlap leaf: L1; fifth visible dewlap leaf: L5; *R*: root. Superscript letters indicate a significant difference between tissues within the same genotype. Significance was calculated via one-way ANOVA, with the post hoc LSD test to separate statistically dissimilar groups. Statistical analysis was measured separately within each genotype

Compiled weight percentages based on five general categories including hemicellulose, cellulose, lignin, extractives and “other”, reported at DW are presented in Fig. 2; Additional file 1: Fig. S1, 2; Tables S2, 3. The other category presumably contains protein, cutins, uronic acids, ash, starch, organic acids and other hydrophobic compounds [30, 37, 38]. The breakdown of the extractives category is determined later in “Soluble sugars”. Overall, cell wall composition variation was observed across organ types and internodal development stage. Hemicellulose and lignin fractions were significantly higher in root (*R*) and leaf (L1 and L5) tissues compared to internode (TI, MI and BI) tissues. Cellulose content was also significantly higher in root (*R*) and leaf (L1 and L5) compared to internode (TI, MI and BI) tissues within the Q208 genotype; however, in KQ228 only mature internodal (BI and MI) tissues had significantly lower cellulose contents compared to leaf (L1 and L5) and root (R) tissues. The extractives fraction was significantly higher in all internode (TI, MI and BI) tissues, in comparison to root (*R*) and leaves (L1 and L5). The “other” fraction was determined significantly higher in leaf tissues (L1 and L5) in comparison to mature internode tissue (MI and BI).

**Table 2:** Syringyl and guaiacyl (S/G) lignin ratios

Genotype	Organ	S/G
KQ228	BI	1.2^a^
	MI	0.7^b^
	TI	0.5^b^
	R	0.7^b^
	L1	0.6^b^
	L5	0.6^b^
Q208	BI	1^a^
	MI	1^a^
	TI	0.4^b^
	R	0.3^b^
	L1	0.3^b^
	L5	0.4^b^

KQ228 and Q208 refer to the *Saccharum* spp. hybrid genotype.

*TI* top internode, *MI* middle internode, *BI* bottom internode; first visible dewlap leaf: L1; fifth visible dewlap leaf: L5; R: root. Superscript letters indicate a significant difference between tissues within the same genotype. Significance was calculated via one-way ANOVA, with the post hoc LSD test to separate groups. Statistical analysis was measured separately within each genotype

**Table 3:** Correlation of cell wall component vs. glucose released from the cell wall during enzymatic saccharification

Saccharified sugar	Biomass component	vs. Sugarcane tissue component	Pearson correlation	Significance (2-tailed)	*R*^2^ (Regression)	Slope (b1)	Significance (Regression)
% Glucose released	Cell wall	Cellulose	− 0.3	0.08	0.3	− 0.08	0.08
		Hemicellulose	− 0.18	0.3	0.03	0.04	0.00
		Lignin	*− 0.66*	*0.00*	*0.44*	*− 0.19*	*0.00*
	Hemicellulose	Mixed-linked Glucan	*0.43*	*0.01*	*0.19*	*0.27*	*0.01*
		Xylose	− 0.07	0.67	0.01	− 0.04	0.67
		Arabinose	*− 0.41*	*0.01*	*0.17*	*− 0.11*	*0.01*
		Galactose	*− 0.45*	*0.01*	*0.2*	*− 0.12*	*0.01*
	Lignin	Guaiacol	0.09	0.6	0.01	0.01	0.6
		Ethyl-2-Phenol	*0.52*	*0.00*	*0.27*	*0.04*	*0.001*
		Creosol	− 0.28	0.11	0.08	− 0.01	0.11
		4-Ethyl guaiacol	0.28	0.10	0.08	0.02	0.1
		4-Vinyl guaiacol	− 0.28	0.1	0.08	− 0.13	0.1
		Syringol	0.24	0.15	0.06	− 0.06	0.15
		Isovanillin	0.18	0.29	0.03	0.02	0.29
		Isovanillic acid	0.23	0.18	0.05	0.07	0.18
		Isoeugenol	*− 0.43*	*0.00*	*0.19*	*− 0.05*	*0.01*
		Methoxy-eugenol	− 0.04	0.8	0.00	− 0.02	0.8
		p-Coumaric acid	0.1	0.56	0.01	0.13	0.56
		Ferulic acid	− 0.15	0.38	0.02	− 0.01	0.38
		AIL	*− 0.67*	*0.00*	*0.45*	*− 1.61*	*0.00*
		ASL	*− 0.6*	*0.00*	*0.36*	*− 0.45*	*0.00*
		S/G ratio	0.05	0.77	0.00	0.00	0.77
	Other analyses	FA/Ara ratio	*0.43*	*0.01*	*0.18*	*0.00*	*0.01*
		Cont. of glucan	0.15	0.39	0.02	0.00	0.39

**Fig. 3:**
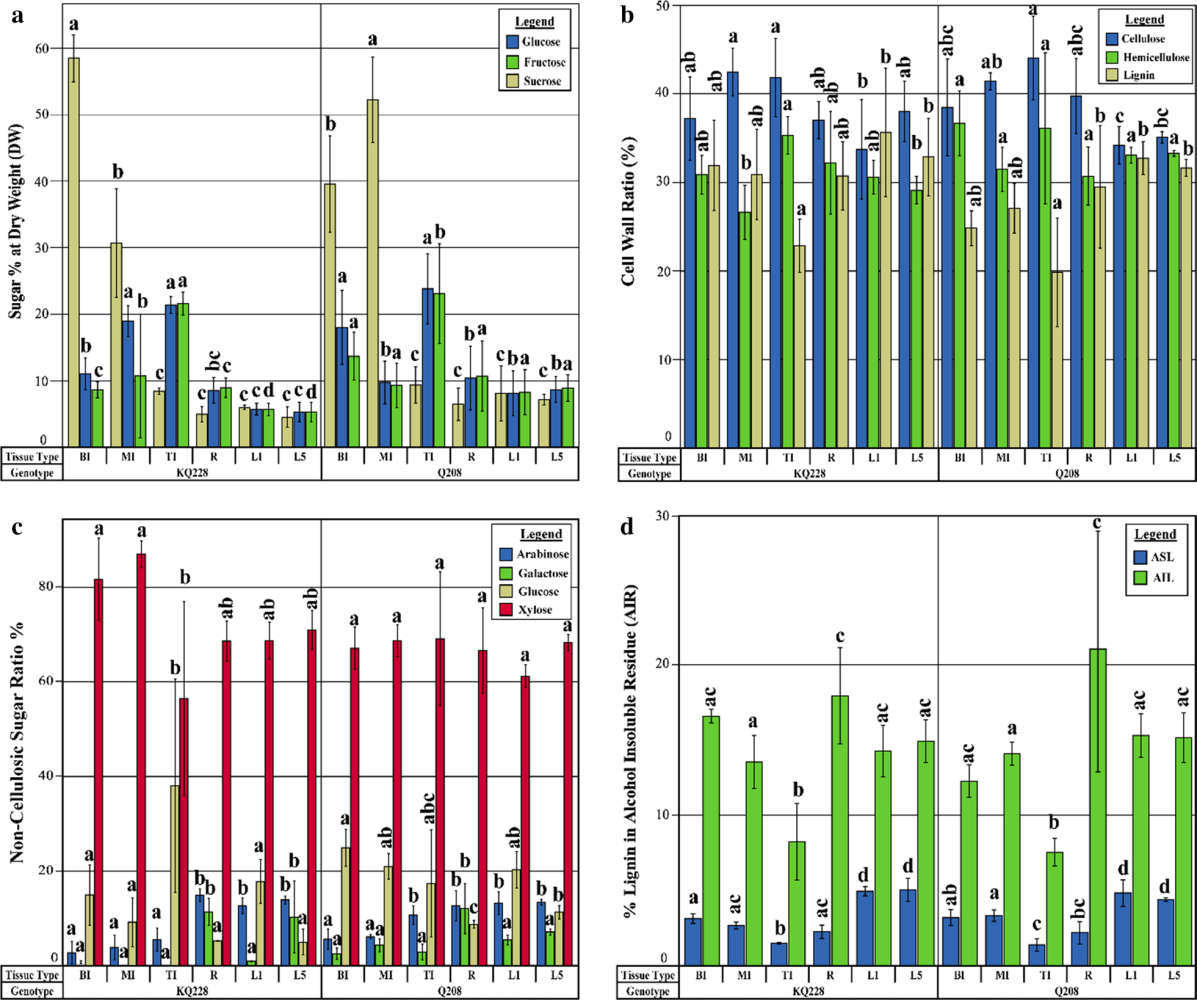
Soluble sugar, cell wall, hemicellulose and lignin contents in sugarcane.** a** Soluble sugar presented as a percentage of dry weight as determined by HPLC. **b** % Ratio of the three major cell wall components. As determined using Klason lignin, HPLC and HPAEC quantification. Values reported as a percentage of total cell wall components. **c** % Ratio of non-cellulosic sugars, otherwise known as mol%, as determined by HPAEC. Values reported as a percentage of total non-cellulosic components. **d** Acid-soluble (ASL) and acid-insoluble (AIL) lignin fractions. As determined in the Klason lignin methodology and subsequent spectroscopy of the hydrolysate. KQ228 and Q208 refer to the *Saccharum* spp. hybrid genotype. Abbreviations, TI: top internode; MI: middle internode; BI: bottom internode; first visible dewlap leaf: L1; fifth visible dewlap leaf: L5; *R*: root. Letters above each bar indicate the presence of a significant difference between values within the same genotype. Error bars ± 1 S.D. from biological triplicates. Significance was calculated via one-way ANOVA, with the post hoc LSD test to separate statistically dissimilar groups. Statistical analysis was measured separately within each genotype

All tissues from both genotypes included in the analysis (*N* = 36). Values compared are presented in Fig. 3. S/G ratio stands for the ratio of syringyl and guaiacyl lignin species, FA/Ara ratio is the ratio of ferulic acid and arabinose in each sample, and cont. of glucan describes the content of glucan/(lignin + xylan), as described in [38, 39, 33], respectively. AIL and ASL stand for acid-insoluble lignin and acid-soluble lignin, respectively. Italic columns display components with a significant |Pearson correlation value|≥ 0.3 and *p* ≤ 0.05

### Soluble C fraction

#### Soluble sugars

The differences in major soluble sugar content between the two sugarcane genotypes and tissue types were determined (Fig. 3a; Additional file 1: Figs. S3, 4; Tables S4, 5). Sucrose content was significantly higher in mature internodal tissues MI and BI in both genotypes than in immature internodal tissue, roots and leaf tissues. In KQ228 genotype, the sucrose content of the BI tissue was significantly higher than that in MI tissue, whereas the opposite was observed in the Q208 genotype, whereby the sucrose content was significantly higher in the MI tissue. Fructose contents were significantly higher in immature internodal tissue in comparison to mature internodes (MI and TI), root and leaf tissues. Also, TI tissues have higher sucrose contents than mature internodal tissues (BI and MI), root and leaf tissues, although in the KQ228 genotype there was no significant difference between TI and MI tissues. Sucrose was the most abundant soluble sugar in mature internodal tissue, whereas glucose and fructose were equally the most abundant in immature internodal tissue. Within leaf tissue, glucose, fructose and sucrose levels were largely equal, whereas in root tissue hexose levels were higher than that of sucrose.

### Insoluble C fraction

#### Cell wall ratio

The ratio of the three major cell wall components, presented as a percentage, in each of the six tissues in both genotypes are presented in Fig. 3b; Additional file 1: Figs. S5, 6; Tables S6, 7. Hemicellulose, cellulose and lignin percentages were deemed to not be significantly different between root, leaf and mature internodal tissues, due to the lack of consistency in results across both genotypes. A significant difference was observed between leaf tissues and immature internodal tissue (TI) across both genotypes, whereby lignin fractions were significantly lower in immature internodal tissue.

#### Non-cellulosic sugar ratio

The determination of released non-cellulosic sugars from the TFA hydrolysis was completed using HPAEC. Four non-cellulosic fractions, mixed-link β-glucan (MLG, named as glucose in table), xylose, galactose and arabinose, are reported as a % of the total non-cellulosic fraction (Fig. 3c; Additional file 1: Figs. S7, 8; Tables S8, 9). Other non-cellulosic components including fucose, rhamnose, galacturonic acid, glucuronic acid and mannose were also quantified; however, their content was too low to be accurately appraised. Arabinose in mature internode (MI and BI) was significantly lower than that of all other tissues in both genotypes. Arabinose was also significantly lower in all internodal tissues in comparison to root and leaf tissues, except for the TI tissue in genotype Q208. Galactose was significantly higher in root tissue than all internodal tissues and leaf tissues; however, no significant difference was detected between root and L5 tissue in genotype KQ228. Xylose and glucose contents whilst displaying some significant differences did not show any consistent organ-specific or maturity-based differences within both genotypes, hence the observations were deemed non-significant.

#### Total lignin content

Acid-soluble lignin (ASL) and acid-insoluble lignin (AIL) were determined using the Klason lignin methodology via spectroscopic and gravimetric means, respectively, reported as a percentage of total AIR (Fig. 3d; Additional file 1: Figs. S9, 10; Tables S10, 11). The larger proportion of the total lignin was acid insoluble. The ASL fraction was significantly higher in leaf tissues in comparison to all other tissues. The AIL fraction was significantly lower in immature internodal (TI) tissues, in comparison to all other tissues. However, in the Q208 genotype, the component was not significantly different between TI and MI tissues. Total lignin was significantly lower in immature internodal tissues in comparison to all other tissues in both genotypes.

#### Lignin compounds

**Fig. 4:**
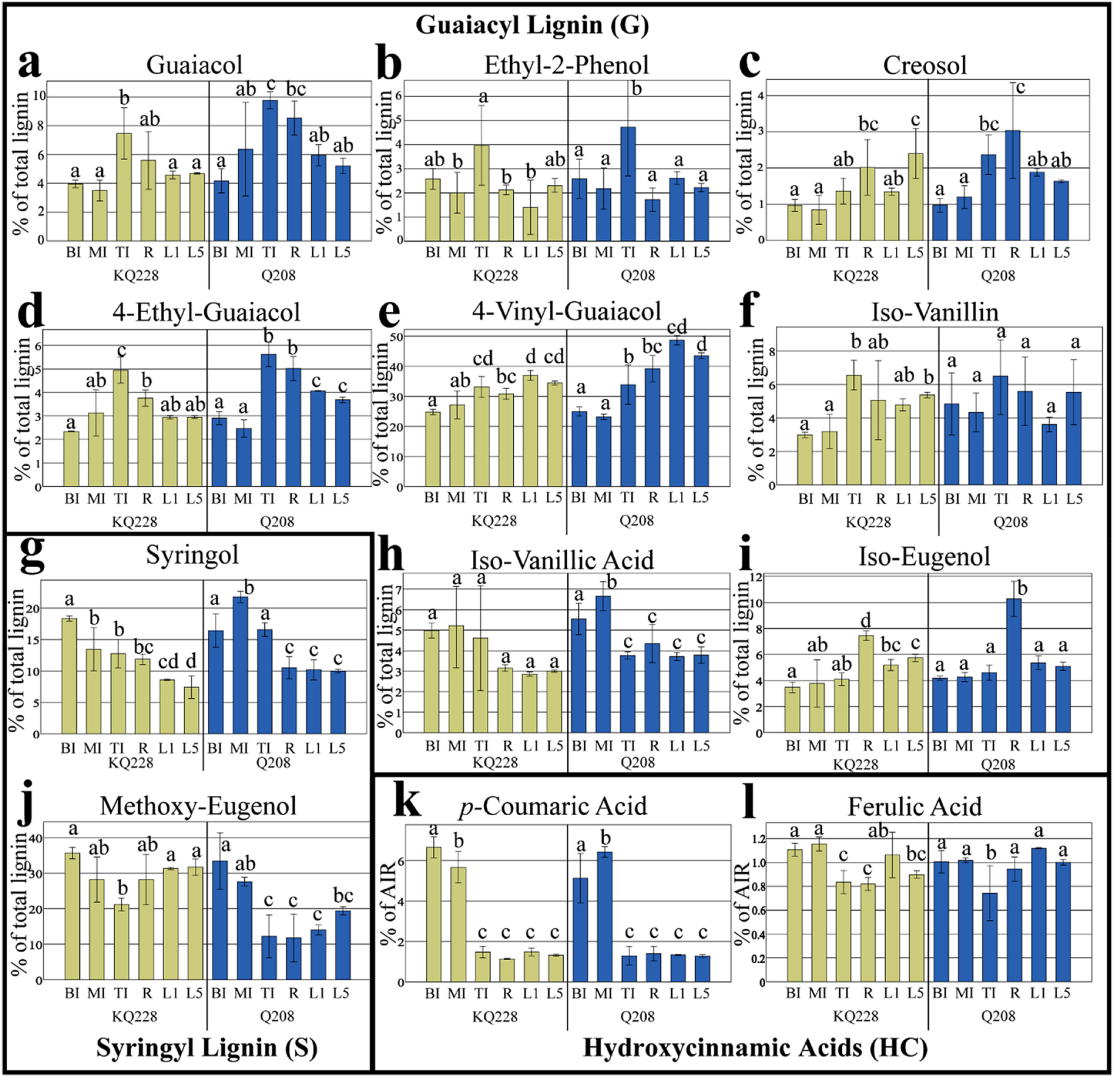
Contribution of lignin-derived compounds in the AIR fraction of *Saccharum* spp. hybrids. Genotype Q208 is demarcated in dark blue, and KQ228 is demarcated in tan. The compounds originate from guaiacyl (G) **(a–e**, **h–i)**, syringyl (S) **(g, j)** and hydroxycinnamic acids (HC) **(k, l)**. Note: hydroxycinnamic acids were detected separately via HPLC determination, and are presented as a % of alcohol-insoluble residue (AIR). Guaiacyl and syringyl monolignols presented as total % of S and G lignin. Abbreviations, TI: top internode; MI: middle internode; BI: bottom internode; first visible dewlap leaf: L1; fifth visible dewlap leaf: L5; *R*: root. Letters above each bar indicate the presence of a significant difference between values within the same genotype. Error bars ± 1 S.D. from biological triplicates. Significance was calculated via one-way ANOVA, with the post hoc LSD test to separate statistically dissimilar groups. Statistical analysis was measured separately within each genotype

Lignin-derived compounds were determined via GC/MS pyrolysis (Fig. 4a–j), whilst hydroxycinnamic acids (HC), *p*-coumaric and ferulic acid were determined via separate hydrolysis and subsequent HPLC determination (Fig. 4k, l). The compounds originated from the two primary lignin monomers guaiacyl (G) (Fig. 4a–f, h–i), and syringyl (*S*) (Fig. 4g, j). Monomers in Fig. 4a–j were determined as the % of the total S and G lignin molecules, whilst monomers k and l were reported as a % of AIR. The ratio of S and G lignin was also calculated (Table 2).

Compounds 4-vinyl-guaiacol, syringol, methoxy-eugenol, *p-*coumaric acid and ferulic acid were the major lignin species detected across all tissues. The HC lignin component *p-*coumaric acid was close to fourfold higher in mature internodal tissues (MI and BI) compared with all other tissues, within both genotypes (Fig. 4k). Ferulic acid composition tended to be lower in immature internodal tissues compared to mature internodes (BI and MI). Further significant differences in ferulic acid content were also observed between TI and L1 tissues of both genotypes (Fig. 4l). Syringol levels were significantly higher in mature internodal tissues than both leaf and root samples in both genotypes (Fig. 4g). Significantly more isoeugenol was observed in root tissues in comparison to all internodal and leaf tissues in both genotypes (Fig. 4i). Guaiacol content was significantly higher in immature internodal tissues in comparison to mature internode (MI and BI) and leaf (L1 and L5) tissues (Fig. 4a.) Methoxy-eugenol content was significantly higher in mature internodal tissues (MI and BI) in comparison to the immature internode (TI), although no significant difference was observed between MI and TI tissues in KQ228 genotype (Fig. 4j). Significantly higher 4-ethyl-guaiacol contents were observed in immature internodal tissue vs. mature internodal tissues in both genotypes (Fig. 4d). All other fractions displayed significance in one or both genotypes between some of the tissue types; however, no specific content pattern was observed, hence they were not described as significant. S/G ratios were significantly higher in mature internodal tissues in comparison to all other tissues, except for MI tissue in KQ228 genotype which had ratios similar to that of root, leaf and immature internodal samples (Table 2). For LSD test values of syringyl, guaiacyl and hydroxycinnamic acid values, see Additional file 1: Fig. S11, Table S12 for KQ228 genotype, and Additional file 1: Fig. S12, Table S13 for Q208 genotype. For LSD test values of S/G ratios, see Additional file 1: Fig. S13, Table S14 for KQ228 genotype, and Additional file 1: Fig. S14, Table S15 for Q208 genotype.

### Enzymatic saccharification

**Fig. 5:**
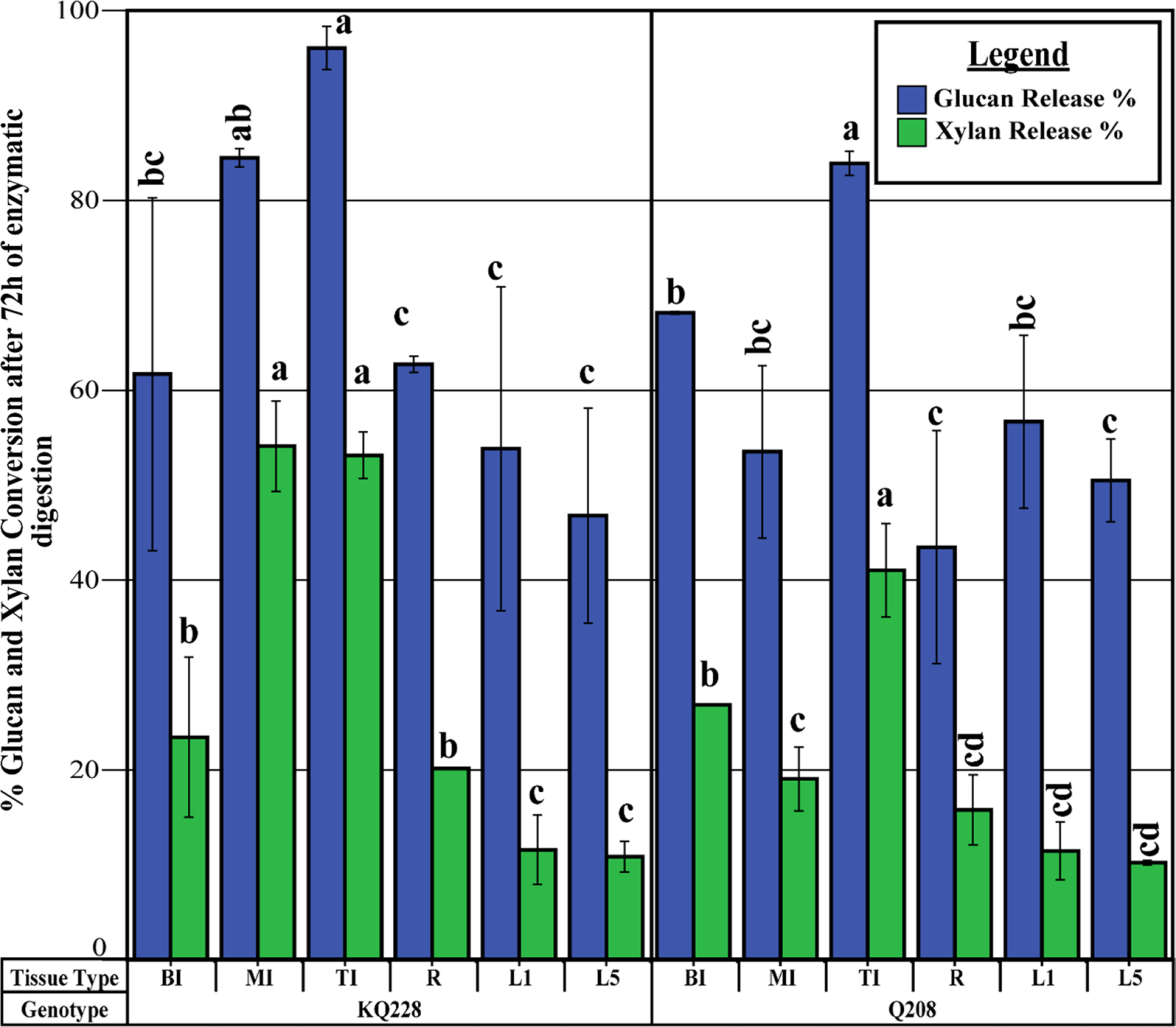
Glucan and xylan conversion after 72 h of enzymatic digestion of the AIR fraction *Saccharum* spp. hybrids. Alcohol-insoluble residue (AIR) subjected to enzymatic digestion by Ctec2:Htec2 enzyme treatment. The percentage value represents the amount of available xylan and glucan (in AIR) released during enzymatic digestion. Abbreviations, TI: top internode; MI: middle internode; BI: bottom internode; first visible dewlap leaf: L1; fifth visible dewlap leaf: L5; R: root. Letters above each bar indicate the presence of a significant difference between values within the same genotype. Error bars ± 1 S.D. from biological triplicate. Significance was calculated via one-way ANOVA, with the post hoc LSD test to separate statistically dissimilar groups. Statistical analysis was measured separately within each genotype

All samples were subjected to enzymatic saccharification over a 72 h period, after which the glucose and xylose released from the cell wall during saccharification were determined via HPLC. The percentage of released xylose and glucose from the available cell wall fraction was determined (Fig. 5 and Additional file 1: Figs. S15, 16; Tables S16 and 17). The amount of glucose released from the cellulose and β-glucan fraction was significantly higher in the TI fraction, in comparison to all other tissues in both genotypes. Further, the MI tissue in KQ228 had significantly higher glucose release than L1, L5 and R tissues. Xylose release from the hemicellulose fraction was also significantly higher in TI internodal tissues in comparison to all tissues across both genotypes, except for MI tissue in genotype KQ228. All internodal tissues had significantly higher xylose release than both leaf tissues in both genotypes, except for MI tissue in Q208 genotype.

#### Regression/correlation analysis

The release of sugars (xylose and glucose) from the AIR (insoluble fraction) was compared to the levels of the insoluble components present using regression and Pearson correlation model calculations across all calculated tissues and biological replicates (Tables 3, 4). Based on the calculated regression and Pearson correlation values, the presence of lignin, particularly the AIL fraction, had the highest negative correlation (− 0.67) with glucose release, closely followed by total lignin (− 0.66). There was no significant correlation with the cellulose and hemicellulose fractions. Within the hemicellulose fraction, MLG content had a moderate significant positive Pearson correlation with total glucose release (0.43), whereas arabinose (− 0.41) and galactose (− 0.45) content had a significant negative correlation with total glucose release. Within the lignin fraction, ethyl-2-phenol has a strong positive correlation (0.52), and isoeugenol had a moderate negative correlation with glucose release (− 0.43). Xylose release also had a high negative Pearson correlation with total lignin content (− 0.65), particularly the ASL fraction (− 0.7). Within the lignin fraction, 4-vinyl-guaiacol (− 0.39) and isoeugenol (− 0.38) had moderate significant negative Pearson correlations with xylose release. There were also lignin components that had a moderate positive correlation with xylose, and this included ethyl-2-phenol (0.41) and isovanillic acid (0.34). Within the hemicellulose fraction, arabinose (− 0.57) and galactose (− 0.5) content had a strong negative Pearson correlation with glucose release. Also, within the hemicellulose fraction, the MLG fraction had a low to moderate positive Pearson correlation with xylose release (0.35).

**Table 4:** Correlation of cell wall component vs. xylose released from the cell wall during enzymatic saccharification

Saccharified sugar	Biomass component	vs. Sugarcane tissue component	Pearson correlation	Significance (2-tailed)	*R*^2^ (regression)	Slope (b1)	Significance (regression)
% Xylose released	Cell wall	Cellulose	0.01	0.9	0.00	0.04	0.94
		Hemicellulose	0.18	0.3	0.00	0.13	0.85
		Lignin	*− 0.65*	*0.00*	*0.42*	*− 1.9*	*0.00*
	Hemicellulose	Mixed-linked Glucan	*0.35*	*0.04*	*0.12*	*0.25*	*0.04*
		Xylose	0.11	0.53	0.01	− 0.07	0.53
		Arabinose	*− 0.57*	*0.00*	*0.33*	*− 0.17*	*0.00*
		Galactose	*− 0.5*	*0.002*	*0.25*	*− 0.16*	*0.00*
	Lignin	Guaiacol	0.13	0.45	0.02	0.02	0.45
		Ethyl-2-phenol	*0.41*	*0.01*	*0.17*	*0.03*	*0.01*
		Creosol	− 0.26	0.124	0.07	− 0.01	0.12
		4-Ethyl guaiacol	0.29	0.091	0.08	0.02	0.09
		4-Vinyl guaiacol	*− 0.39*	*0.02*	*0.16*	*− 0.2*	*0.02*
		Syringol	0.32	0.06	0.1	0.09	0.06
		Isovanillin	0.11	0.5	0.01	0.01	0.5
		Isovanillic acid	*0.34*	*0.04*	*0.12*	*0.11*	*0.04*
		Isoeugenol	*− 0.38*	*0.02*	*0.14*	*− 0.05*	*0.02*
		Methoxy-eugenol	− 0.03	0.85	0.00	− 0.02	0.85
		p-Coumaric acid	0.23	0.17	0.01	0.13	0.56
		Ferulic acid	− 0.13	0.44	0.02	− 0.01	0.38
		AIL	*− 0.55*	*0.00*	*0.45*	*− 1.61*	*0.00*
		ASL	*− 0.7*	*0.00*	*0.36*	*− 0.45*	*0.00*
		S/G ratio	0.09	0.62	0.00	0.00	0.77
	Other analyses	FA/Ara ratio	0.27	0.11	0.18	0.00	0.01
		Cont. of glucan	0.3	0.08	0.09	0.00	0.08

All tissues from both genotypes included in the analysis (*N* = 36). Values compared are presented in Fig. 3. S/G ratio stands for the ratio of syringyl and guaiacyl lignin species, FA/Ara ratio is the ratio of ferulic acid and arabinose in each sample, and cont. of glucan describes the content of glucan/(lignin + xylan), as described in [38, 39] and [33], respectively. Italic rows display components with a significant |Pearson correlation value|≥ 0.3 and *p* ≤ 0.05

## Discussion

**Fig. 6:**
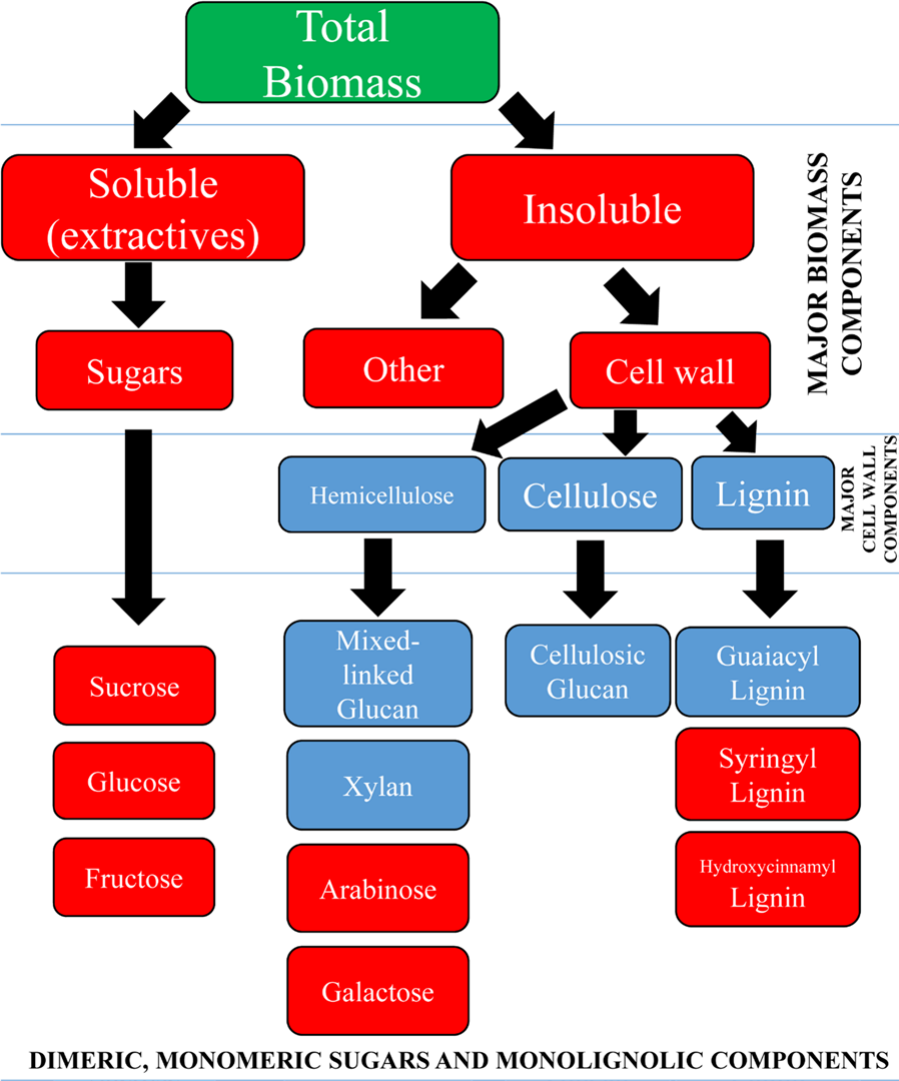
Major components of sugarcane biomass that differed significantly between sugarcane tissue types, as determined in this study. Blue indicates there was no significant difference between leaf, internode and root tissues. Red indicates significant differences between the tissues (*p* ≤ 0.05)

Commercially, the soluble fraction (in the form of sucrose) of sugarcane is utilised in commercial sugar production, whereas the insoluble fibre fraction can be used in several other processes, including electricity co-generation, biofuels, and a variety of biomaterials [2, 40]. In the past decade, utilisation of the insoluble fraction of sugarcane is seen as an ideal feedstock to produce energy products, polymers and non-fossil-based chemicals together within a biorefinery system [9, 41]. Creating more sugarcane fibre that is easier to break down will be key to producing a viable biomaterials sector in the sugarcane industry [9]. Due to the economic importance of the sugarcane culm, the composition of individual internodes has been well characterised between varying levels of maturity and between genotypes [16, 25, 30–33]. The composition in the other important organs of commercial sugarcane, i.e. roots and leaves, has not been characterised to the same extent as internodal tissues. This limits our understanding of compositional differences between different parts of the sugarcane plant, which in terms of total biomass produced are proportional to the culm [34]. Differences in composition between the major organs of sugarcane may have implications regarding their use in specific biorefinery applications. Significant difference was observed between the different sugarcane organ types within the various insoluble and soluble attributes, as summarised in Fig. 6.

### Internodes

Composition of soluble and insoluble fractions varied greatly between immature and mature internodes. MI and BI tissues did not differ significantly in their ratios of soluble and insoluble components. This result was expected, as MI tissue (internodes 11,12 and 13) is fully mature, and hence the high levels of sucrose [25, 28]. Although the total soluble sugar content was similar between immature (TI) and mature internodes (MI and BI), hexoses dominated in the immature tissue and sucrose in the mature tissue, as has been previously described [23, 29, 42]. The dichotomy of soluble sugar content between immature and mature internodes is indicative of the differences in C utilisation within these tissues. Within the immature internodes, heightened fructose and glucose levels indicate an increase in hydrolysis and cleavage of sucrose, indicative of a meristematic sink [43]. The significantly higher “other” fraction and lower ASL and AIL lignin (in AIR) determined in the immature internodes, in comparison to mature internodes is also supportive of this, as a large amount of C moves into protein and cell wall fractions, which is supportive of results in previous studies [25, 33]. Conversely, the lower comparative hexose content in mature internodes displays the bias of C partitioning away from respiration, cell wall and protein, to that of sucrose storage, as has been described in a previous study [16].

Interestingly, the ratio of S/G lignin and the HC lignin compound *p-*coumaric acid was significantly higher in mature internodes. This result suggests that the deposition of S and HC lignin compounds is prevalent in the latter stages of internodal maturation. The difference in S/G ratio between the rind and pith in [44] is similar to the difference in S/G ratio between mature internodal tissues, and mature leaf and root tissues in this study. The biggest differences in the lignin composition were that of the hydroxycinnamic acid, *p-*coumaric acid, within mature internodal tissues. The domination of *p-*coumaric acid over the other common hydroxycinnamic acid, ferulic acid, is characteristic of sugarcane internodal tissues [45, 46]. It has been suggested the *p-*coumaric acid fraction is esterified to arabinoxylans in small amounts during the early stages of tissue development, then, later on, is esterified more extensively into the lignin fraction [47, 48], as reviewed by [49].

The results from this study and data presented in other studies support the notion that C flow into the three major cell wall fractions is highly stable between different organs or cell types in sugarcane [19, 36, 50]. This lack of difference between the major mature organ types of sugarcane (i.e. MI, BI, R, L1 and L5 tissues), as presented in this study, suggests C partitioning to the cell wall occurs in a fixed ratio (Fig. 4b) regardless of differences in related gene expression, enzymatic activity or metabolic conditions between each organ type during cell wall deposition.

### Leaves

Biomass composition of L1 and L5 tissues did not differ in this study. In comparison to mature internodal tissues, C partitioning in leaf tissues as distinguished by the ratio of insoluble and soluble compounds at DW is characterised by low levels of solubles, high amounts of cell wall compounds and ‘other’ compounds. Regarding the ‘other’ compounds, a large degree of this is likely protein, due to the presence of photosynthetic machinery within leaf tissues. Characterisation of protein in sugarcane tops, which is largely made up of photosynthetically active leaves supports this notion, with up to 7% protein found within this tissue [17]. Furthermore, the low amount of soluble sugars in leaf tissues likely indicates the synthesis and transportation of sucrose to sink tissues [51]. Analysis of the cell wall % ratio of the three major cell wall components displayed no difference vs. that of mature internodal tissues. This observation further supports the hypothesis that the ratio of C partitioned into the cell wall fraction is tightly fixed. Alternatively, within the hemicellulose and non-cellulosic fraction, significantly higher levels of arabinose and galactose were observed in leaf tissues vs. internodal tissues, which shows the monomeric makeup of the non-cellulosic fraction to vary between tissues, as has been shown in another study [36].

### Roots

The low levels of sucrose in sugarcane root tissue display that there is no active C storage, as sucrose, occurring in this tissue. Immature internodal tissue is presumably like root tissue in its utilisation of C, as they both likely assimilate the bulk of C into the cell wall, protein and respiratory pools. The hexose–sucrose profile observed in root tissue (i.e. lower sucrose and higher reducing sugars) was similar to that of immature internodal tissue. However, significantly lower levels of sucrose, fructose and glucose were observed in root tissue in comparison to the immature internodal tissue. This result suggests an enhanced capacity for the root tissue to utilise sucrose and reducing sugars, in comparison to immature internodes. Within immature internodes, a significant portion of sucrose hydrolysis occurs in the vacuole, with up to 95% of reducing sugars being found within [42]. This means metabolism/utilisation of said reducing sugars is restricted by the movement out of the vacuole. The lower reducing sugar content in the root tissue suggests that sucrose hydrolysis does not occur within the vacuole, instead likely occurs within the cytosol or intercellular space, as per [52].

Within the less abundant neutral sugar subunits from the non-cellulosic fraction, significant differences were observed between roots and internodal tissues suggesting differences in mixed-linkage substitution and/or the differential presence of pectins. The high galactose content in the roots compared with the other tissues may display differential pectin deposition between tissues. This is further supported by the significantly higher arabinose contents also found in the root tissues in comparison to mature internodal tissues in this study, which commonly form pectic complexes [19, 53]. As grass cell walls primarily contain arabinoxylans with insignificant amounts of pectic polysaccharides, it could be assumed that most of the arabinose goes into the arabinoxylan fraction. However, the heightened arabinose and galactose content in root tissue may represent the pectin fraction in this tissue, which is not present in internodal tissues. Significantly higher arabinose contents found in leaf tissues in comparison to internodal tissues could also represent this pectic fraction; however, the galactose fraction in most leaf samples was not significantly higher, which could suggest differences in pectic content. It is also possible that the increased arabinose fraction represents an increase in arabinan substitution in both root and leaf tissues [36].

### Enzymatic saccharification

The effect of biomass composition on recalcitrance in different sugarcane organs was measured by subjecting samples (AIR) to brief autoclaving followed by enzymatic hydrolysis for 72 h [54]. The rate of saccharification differed greatly between different sugarcane tissue types and between genotypes, suggesting the interaction of several different compositional elements. Based on the regression analysis of the % of released xylose and glucose from saccharification, we obtained cell wall attributes of interest that correlate relatively tightly with recalcitrance. Multiple studies have proposed numerous biomass features that contribute to biomass recalcitrance [19, 20, 33]. Unfortunately, the published literature is often conflicting, and as a result, no clear picture has emerged as to what plant features most strongly limit efficient sugar release [55].

Total lignin was found to have a large negative effect on xylose and glucose release. This finding agrees with current literature, suggesting total lignin content is one of the most important factors in recalcitrance [56–58]. In contrast with previous studies [20, 33], no clear correlation was observed between glucan (cellulose + MLG) content and glucose release. This may be due to the positive correlation between cellulose and lignin content in these samples. Additionally, differences in biomass pretreatment before enzymatic saccharification, the tissues/organs analysed, and the specific biomass values (i.e. values in AIR, DW or as a molecular ratio) likely contributed to this difference. It should be noted that within the hemicellulose fraction, MLG content was moderately positively correlated with glucose and xylose release, which agrees with the aforementioned studies. Individual S and G lignin monomers were found to have moderate to high correlations with both xylose and glucose release. S and G units amalgamate to form the backbone of the lignin polymer via a labile-aryglycerol-β-aryl ether (β-*O*-4) bond [59]. The strong correlation of individual S and G lignin units was not backed by a highly correlated S/G ratio. The S/G ratio is in many cases highly conflicting as evidenced by the multitude of studies that have found very strong [60–63] and very weak [57, 63] correlations. However, it is possible that the high variability in monolignol content across tissues affected the correlation in this case. Correlations between individual S and G lignin monolignols and biomass recalcitrance have not yet been investigated in sugarcane. Arabinan and galactan were found to have a clear negative correlation with xylose and glucose release. Arabinose substitution into arabinoxylan complexes is well known to greatly increase biomass recalcitrance [36]. Additionally, hemicellulosic polysaccharides with additional branching are well known to have a significantly higher resistance to saccharification than less decorated polymers [55, 64], which explains the negative correlation between both arabinose and galactose content on xylose release. In individual tissues/organs, the observed higher enzymatic digestibility of immature internodes agrees with a previous study [33]. The aforementioned study referenced the low lignin levels in immature internodes as a core factor in the observed high digestibility. The tendency for higher digestibility in all internodes vs. leaf tissues contradicts a previous study in sugarcane that displayed the opposite trend, albeit in whole internode and whole leaf comparison [20].

## Conclusion

The major organs of sugarcane are distinctly different compositionally. Distinct compositional differences were identified throughout six sugarcane tissues in two commercial genotypes. Overall, the organ-specific analysis presented helps to define the partitioning of carbon into the major soluble and insoluble fractions between the three major sugarcane organs, root, leaf and internodes. Whilst no differences were observed in the ratio of major biomass fractions (hemicellulose, cellulose and lignin) between the root, leaf and mature internodal tissues, clear compositional differences were identified within the monomeric fractions of these traits. This was particularly the case within the hemicellulose and lignin fractions, suggesting differential control of these fractions between each organ type. Enzymatic saccharification analysis revealed the total lignin fraction, arabinose and galactose to be the leading factors in biomass recalcitrance. The results in this multifaceted analysis can be used to inform future genetic studies, and as a potential reference for future genetic manipulation to produce sugarcane genotypes with optimised biomass compositions for a multitude of biofuel and biomaterial applications.

## Materials and methods

### Plant material

Tissue used in this experiment is derived from two commercial sugarcane varieties, KQ228 and Q208. Sample material was provided by Sugar Research Australia (SRA), which was grown at their station in Brandon, Queensland, Australia. Leaf and internode samples of both genotypes were taken from a 10-month-old commercial stands. Field-grown material contained between 24 and 25 internodes, at an average height of 2.7 m, from the base of the stem to the top of leaf roll. Root samples of Q208 and KQ228 were obtained from 3-month-old research plants in ‘soft’ aboveground pots. 3-month-old plants contained nine internodes, at an average height of 50 cm. All samples were collected in triplicate.

### Sugarcane material collection

All samples were ‘snap-frozen’ in liquid nitrogen within a minute of excision, put on dry ice in transit and kept in a − 80 °C freezer before preparation. Root material was taken 20 cm away from the base of the stem; see Fig. 1. Roots (*R*) were removed from the pots, washed with a fine mist spray nozzle, and contact dried with a paper towel before snap freezing. Roots were frozen within 30 s of removal from soil. Leaf material was obtained from the first and fifth leaves with visible dewlaps (L1 and L5, respectively); see Fig. 1. Internodes were numbered following McCormick et al*.* [43] proposed a schematic. Immature internode (labelled as “top internode”, TI) material was collected from the second, third and fourth internodes. Mid-range mature internode (labelled as “middle internode”, MI) samples were collected from the 11th, 12th and 13th internodes. Bottom-range mature internode (labelled as “bottom internode”, BI) material was collected from the 21st, 22nd and 23rd internodes. Internode samples were taken as small disks 0.5 cm in height, which were processed into small cubes before snap freezing. Schematic of the tissues sampled is shown in Fig. 1a.

### Sample preparation

Samples were prepared based on the National Renewable Energy Laboratory (NREL) method (Lyophilisation) in *“Preparation of Samples for Compositional Analysis”* [65], at the University of Queensland, St Lucia, QLD, Australia*.* Samples were homogenised in cryogenic conditions using a Retsch TissueLyser™ II (Retsch, Haan, Germany) at a frequency of 30/S for 1.5 min. Following homogenisation, samples were weighed then lyophilised in a VirTis Benchtop™ “K” series freeze dryer (VirTis, Gardiner, NY, USA). The sample weight was recorded after 3 days in the lyophiliser, then the samples were reweighed after an additional day of lyophilisation to ensure all water was removed from samples. The sample material was weighed before and after freeze-drying to determine the water content. The freeze-dried samples were sent via FedEx International Priority to the facilities at the Joint Bioenergy Institute (JBEI), Emeryville, CA, USA.

### Alcohol-insoluble residue (AIR) preparation and de-starching

AIR was prepared using a modified method described in [66]. Due to the high sugar content of some of the sugarcane samples, particularly the bottom and middle internodes, the AIR preparation protocol was extended to ensure no soluble sugars were retained. The extended protocol was as follows: 300 mg of pre-dried tissue was added to a 50 mL Falcon tube, followed by the addition of 40 mL of 100% (v/v) ethanol. The AIR/ethanol solution was mixed using a Fisher Scientific Vortex-Genie 2™ (Fisher Scientific, New Hampshire, USA) for 30 s. The solution was then centrifuged in an Eppendorf Centrifuge 5810R™ (Eppendorf, Hamburg, Germany) at 4000*g*, at room temperature (RT) for 15 min. Following centrifugation of 2 mL of the ethanol, extractive supernatant was collected and put into a separate labelled 50 mL Falcon tube, the remaining ethanol supernatant was discarded. The previous steps were repeated with the following washes (in order): methanol:chloroform (2v:3v; equal to 16 mL of methanol and 24 mL of chloroform), methanol:chloroform (same volumes as 1^st^ wash), 100% ethanol, 65% ethanol, 80% ethanol, 80% ethanol, 100% ethanol and 100% ethanol. An 18 mL aliquot of alcohol extractives solutions was retained for soluble sugar analysis. Following the final ethanol wash, the resulting AIR was de-starched, using consecutive enzyme incubations of thermostable α-amylase and an amyloglucosidase and pullulanase mixture, respectively, in 45 mL of 10 mm potassium phosphate buffer. All enzymes were added to the AIR/buffer solution at a rate of 1 enzymatic unit/mL. Further details of the de-starching method are outlined in [67]. Starch, as a polymer of glucose, can disrupt the accuracy of downstream cell wall analysis, disrupting the accuracy of the hydrolysed glucose yield from hemicellulose and cellulose [68]. Following de-starching, samples were lyophilised over 2 days in a Virtis™ benchtop freeze drier, until a stable weight was obtained.

### High-performance liquid chromatography (HPLC) of AIR

A 2 mL aliquot of the 18 mL ethanol extractives supernatant derived from the AIR preparation protocol was dried down under nitrogen (N) gas in a Techne Sample Concentrator (Techne, Charleston, SC, USA). The extractives precipitate was resuspended in 200µL of Milli-Q^®^ water (18.2 Ω), filtered through a 0.45 µm filter plate, and then loaded into the high-performance liquid chromatography (HPLC) system. The samples were run on an Agilent 1260 Infinity™ LC series system (Agilent, Santa Clara, CA, USA), with a Bio-Rad 300 × 7.8 mm Aminex 87 H column (Bio-Rad, Hercules, CA, USA) and a Bio-Rad cation H guard column. The Agilent 1260 refractive index detector (RID) was held at 35 °C. The samples were run using an isocratic 4 mM sulphuric acid eluent at 0.6 mL/min^−1^ and 60 °C for 16 min. For the quantification of sucrose, glucose, and fructose, the conditions using a temperature of 18 °C and 10 mM sulphuric acid eluent at a flow rate of 0.3 mL/min^−1^ for 22 min. The extractive precipitate sample volume was 3µL (at RT). A combined sucrose, fructose and glucose standard from Sigma-Aldrich (St Louis, MI, USA) at concentrations of 5, 10, and 20 g/L was run as a signature reference, which displayed satisfactory accuracy of the HPLC instrument. For further details of the methods and instruments utilised, see [69].

### Determination of hemicellulose, cellulose, lignin and insoluble ash in AIR

Basic compositional analysis of AIR was conducted for the major lignocellulosic components cellulose, hemicellulose, lignin and ash, using NREL methods, altered for sugarcane fibre [66]. 100 mg of AIR underwent acid hydrolysis using 1 mL of 72% sulphuric acid (H_2_SO_4_), subjected to a 1 h incubation at 30 °C. Following incubation, the sulphuric acid (SA) was diluted to 4% with Milli-Q^®^ water (18.2 Ω), then autoclaved for 1 h at 121 °C. Cellulose and hemicellulose hydrolysate analysis in HPLC was undertaken using the SA hydrolysate supernatant following autoclaving. A 2 mL aliquot of the diluted sulphuric acid supernatant was dried down using N gas, resuspended in 200 µL of milli-Q water and filtered through 0.45 µm filter. SA supernatant was run on an Agilent 1260 Infinity™ LC series HPLC system, through a Bio-Rad 7.8 mm x 300 mm Aminex 87H column (Bio-Rad, Hercules, CA, USA), with a Bio-Rad cation H guard column. The Agilent 1260 refractive index detector (RID) was held at 35 °C. The samples were run using an isocratic 4 mM sulphuric acid eluent at 0.6 mL/min^−1^ and 60 °C for 16 min. Sugar calibration standards were prepared and diluted to create six-point calibration curve, 0.0156—2.0 mg/mL for xylose and 0.03125–4.0 mg/mL for glucose, and 0.325–20 mg/mL for glucose. For further details regarding methods and instruments utilised, see [70]. Acid-soluble lignin (ASL) was determined using a 2 µL aliquot of the filtered supernatant solution, on a Thermo Scientific NanoDrop™ 2000 (Thermo Fisher Scientific, Waltham, MA, USA) UV–visible spectrometer, at 320 nm. The remaining dilute SA supernatant was filtered through a 25 mL vacuum crucible (CoorsTek #60,531) and then dry crucibles (in oven) at 105 °C for a minimum of 6 h. Crucibles were weighed before and after SA supernatant filtration (weighed after oven drying) to calculate the % total of ASL, hemicellulose and cellulose. Following weighing, the crucibles were placed in a Themolyne™ industrial benchtop furnace (Thermo Fisher Scientific, Waltham, MA, USA) at a 575 °C cycle for 6 h. Crucibles were weighed following pyrolysis.

### *Non-cellulosic monosaccharide content determination *via* TFA hydrolysis*

Glucose is a product of both cellulose and hemicellulose hydrolysis. To determine the contribution of both polysaccharide groups to hydrolytic glucose, trifluoroacetic acid (TFA) hydrolysis followed by Saeman hydrolysis was employed, optimised for sugarcane from [71]. The TFA hydrolysis method is as follows: 1 mg of AIR was resuspended in 1 mL of Milli-Q^®^ water (18.2 Ω), then a 50 µL aliquot was taken for downstream analysis, in a 1.5 mL screw cap tube. The 50 µL aliquot was then dried down in an Eppendorf Vacufuge^®^ plus at the following settings: 1 h, 45 °C, V, AQ setting. Following drying, 400 µL of 2 M TFA was added to the dried material, then incubated at 120 °C for 1 h in a Techne Dri-Block^®^ DB-3D. The TFA solution was then centrifuged in an Eppendorf Centrifuge 5418D, at the following settings 4 °C, 10 mins, and 14000*g* (max RPM). The supernatant was removed from the solution and retained in a new 1.5 mL tube. The remaining crystalline pellet was washed with 400 µL of Milli-Q^®^ water (18.2 Ω), vortexed for 30 s, and then centrifuged (using the same settings as above). The supernatant was removed from the milli-q/pellet solution, then pooled with the TFA supernatant. The remaining crystalline cellulose pellet was then dried down in the Vacufuge at the following settings: 1 h, 45 °C, V, AQ setting. The dried crystalline cellulose pellet was retained for Saeman hydrolysis, for glucose determination via HPLC. The combined TFA/Milli-Q supernatant was also dried down at the following settings: RT, ∞ time, V, AQ. Following drying, the supernatant precipitate was resuspended in 200 µL of Milli-Q^®^ water (18.2 Ω). 150 µL of the resuspension was filtered (0.45 µm) then run on a Dionex™ ICS-5000^+^ SP (Thermo-Scientific, Dionex, Sunnyvale, California USA) high-performance anion-exchange chromatograph with pulsed amperometric detection (HPAEC-PAD). A CarboPac PA20 analytical anion exchange column (3 mm x 150 mm; Thermo Fisher Scientific), PA20 guard column (3 mm x 30 mm), and a borate trap was utilised within the HPAEC. Proceeding a 5 min equilibration, the injected sample was eluted using an isocratic gradient of 4 mM NaOH from 0 to 6 min, followed by a linear gradient of 4 mM NaOH to 1 mM NaOH from 6 to 19 min. At 19.1 min, the gradient was increased to 450 mM NaOH to elute the acidic sugars. HPAEC method was based on [72]. Equivalent sugar standards of glucose, xylose, arabinose, galactose, mannose, fucose and rhamnose were utilised.

### Determination of hydroxycinnamic acids

30 mg of AIR was used to determine the ferulic and *p-*coumaric acid content. The AIR was added to a 1.2 mL tube and then suspended in 500 µL of 2 M NaOH. The NaOH suspension was incubated for 24 h at 30 °C. Following incubation, the NaOH solution was neutralised to a pH of 2 using 100 µL of 12.39 M hydrochloric acid (HCl) (Sigma Aldrich), after which 500 µL of ethyl acetate was added. The solution was vortexed (Vortex-Genie 2™) for 30 s and then centrifuged (Eppendorf 5810R) for 5 min at *g* × 4000. After centrifugation, the separated top layer of the supernatant was transferred to a new 1.2 mL tube. The ethyl acetate wash was completed two more times (the top layers from the 3 ethyl acetate washes were combined). The top layer supernatant was then dried down under N gas (Techne Sample Concentrator) and then resuspended using 200 µL in acetonitrile (vortexed for 30 s). Before injection into the HPLC instrument, the solution was filtered (0.45 µm). Standards for coumaric and ferulic acid (Sigma-Aldrich) (CAS No. 501-98-4 & 537–98-4) at 1, 0.5, 0.25, 0.12, and 0.0625 g/L were used as reference. HPLC separation of lignin-derived aromatics (coumaric acid and ferulic acid) was performed on a C18 column (Agilent Zorbax Eclipse XDB-C18) using acetonitrile–water (20:80, containing 0.5% acetic acid) eluent at 1 mL/min and 25 °C for 12 min. Diode array detection (DAD) was performed at 310 and 320 nm.

### GC/MS pyrolysis

AIR pyrolysis in conjunction with gas chromatography–mass spectroscopy (GC–MS) was utilised to semi-quantitatively compare the amount of S and G lignin molecules within each sample. Methods were completed as described in [73]. The samples were pyrolyzed at 550 °C using a CDS analytical Pyrophobe 5200™ (CDS Analytical Inc. Oxford, PA, US) connected to an Agilent 6890 GC–MS system. The GC–MS systems were equipped with a Thermo Electron Trace gas chromatograph (GC) Ultra and Polaris-Q mass spectrometer (MS) (Thermo Electron Corp, Waltham, MA, US) equipped with a TR-SMS column (60 mm × 0.25 mm) operated in the split mode (40 mL/min^−1^) using helium (*He*) as a carrier. The GC was set as per the following: initial temperature of 50 °C for 5 min, then increased by 5 °C increments per min to 300 °C, which was held for 5 min. Organic compounds were identified based on their mass spectra and GC retention time using the NIST spectroscopy data centre (NIST08). S and G molecules were identified based on their specific m/z value (mass/ion charge no.) and quantified as a percentage of the Pyrogram. Relative amounts of S and G molecules were presented as a percentage of the Pyrogram, hence making the calculation semi-quantitative [74]. Specific pyrolysis fragments, their elution time and their origin are listed in Table 5. S/G ratio was calculated as the sum of all peak areas of S molecules divided by the total peak areas of G molecules.

**Table 5:** Compounds found by Pyro GC/MS with elution time and their origin

Compound	Elution time	Origin
Guaiacol	5.64	G
phenol 2-ethyl	8.3	G
2-Methoxy-4-methyl phenol (creosol)	9.55	G
4-Ethyl-guaiacol (4-ethyl-2-methoxy phenol)	12.85	G
4-Vinyl-guaiacol (2-methoxy-4 vinyl phenol)	13.86	G
2,6-Dimethoxy phenol/syringol	14.72	S
3-Hydroxy-4-methoxy benzaldehyde	15.73	G
3-Hydroxy-4-methoxy benzoic acid (isovanillic acid)	16.52	G
Isoeugenol	16.63	G
4-Allyl-2,6-dimethoxy phenol (4- allyl syringol) (cis)	18.8	S

Origin refers to the lignin type, *G* guaiacyl and *S* syringyl

### Enzymatic saccharification

The differing compositional attributes of organs within sugarcane contribute to differences in susceptibility to enzymatic saccharification. The effect of biomass composition on recalcitrance in different sugarcane organs was measured by subjecting samples (AIR) to brief autoclaving, followed by enzymatic hydrolysis for 72 h. Methods were based on a modified protocol outlined in [75]. Saccharification was undertaken with two separate enzymatic mixes with high hemicellulasic and cellulasic qualities: Novozyme Cellic^®^ CTec2:HTec2 (Novozymes, Bagsværd, Copenhagen, Denmark). Xylose and glucose released from the AIR fraction was quantified using HPLC, as previously described. Details of enzymatic dilutions, solution concentrations and total solution size used in the enzymatic saccharification are described in Table 6.

**Table 6:** Details of enzymatic dilutions, solution concentrations and total solution size, used in the enzymatic saccharification

Component	Final concentration	Enzymatic master mix for 36 samples	Total in 1000 µL reaction
Water for hydrothermal pretreatment	–	–	340 µL
Biomass	10 mg/ml (solution)	–	10 mg
50 mM citrate buffer (pH 4.8)	50 mM	29.23 mL	649.468 mL
CTec enzyme	9 enzymatic units per gram of AIR	21.546 µL	0.0532 µL
HTec enzyme	1 enzymatic unit per gram of AIR	2.394 µL	0.4788 µL

### Data processing

All data analysis and illustration were done using XLSTAT ver 2017.7 in Microsoft Excel 2016, RStudio ver 1.1.383 [76] in R ver 3.4.3 [77], and IBM^®^ SPSS^®^ statistics ver. 23 [78]. The statistical significance of the mean of the biological replicates was calculated utilising the one-way ANOVA function, with the additional least significant difference (LSD) post hoc test, within SPSS. The null hypothesis was accepted at a *p* value of ≤ 0.05. Minimum and maximum outliers (two-sided) were removed from some datasets using the modified Thompson Tau test [79]. Regression analysis was undertaken using the dot plotting function in SPSS with the *R*^2^ calculated, to define the best correlations between glucose and xylose release from the cell wall structure and biomass traits, as is commonly used in biomass determination studies [80–82]. The significance of the regression values was calculated using the bivariate correlation function in SPSS.

## Supplementary information

**Additional file 1: Table S1**. Numerical Identifier for LSD post hoc test results. **Figure S1**. Screenshot of Tables presenting LSD post-hoc testing of major biomass fractions in KQ228 genotype. The results relate to figure 2. Screenshots derived from SPSS statistical package. Refer to Table 1 for numbers corresponding to tissue type. Abbreviations, TI: Top Internode; MI: Middle Internode; BI: Bottom Internode; 1st Visible Dewlap Leaf: L1; 5th Visible Dewlap Leaf: L5; R: Root. **Table S2**. Homogenous subsets of major biomass fractions in KQ228 genotype as calculated by LSD post-hoc testing. The results relate to figure 2. Letters indicate the presence of a significant difference between values within the same genotype. **Figure S2**. Screenshot of Tables presenting LSD post-hoc testing of major biomass fractions in Q208 genotype. The results relate to figure 2. Screenshots derived from SPSS statistical package. Refer to Table 1 for numbers corresponding to tissue type. Abbreviations, TI: Top Internode; MI: Middle Internode; BI: Bottom Internode; 1st Visible Dewlap Leaf: L1; 5th Visible Dewlap Leaf: L5; R: Root. **Table S3**. Homogenous subsets of major biomass fractions in Q208 genotype as calculated by LSD post-hoc testing. The results relate to figure 2. Letters indicate the presence of a significant difference between values within the same genotype. **Figure S3**. Screenshot of Tables presenting LSD post-hoc testing of soluble sugar fractions, fructose, glucose and sucrose in KQ228 genotype. The results relate to figure 3a. Screenshots derived from SPSS statistical package. Refer to Table 1 for numbers corresponding to tissue type. Abbreviations, TI: Top Internode; MI: Middle Internode; BI: Bottom Internode; 1st Visible Dewlap Leaf: L1; 5th Visible Dewlap Leaf: L5; R: Root. **Table S4.** Homogenous subsets of soluble sugar fractions, fructose, glucose and sucrose in KQ228 genotype as calculated by LSD post-hoc testing. The results relate to figure 3a. Letters indicate the presence of a significant difference between values within the same genotype. **Figure S4. **Screenshot of Tables presenting LSD post-hoc testing of soluble sugar fractions, fructose, glucose and sucrose in Q208 genotype. The results relate to figure 3a. Screenshots derived from SPSS statistical package. Refer to Table 1 for numbers corresponding to tissue type. Abbreviations, TI: Top Internode; MI: Middle Internode; BI: Bottom Internode; 1st Visible Dewlap Leaf: L1; 5th Visible Dewlap Leaf: L5; R: Root. **Table S5**. Homogenous subsets of soluble sugar fractions, fructose, glucose and sucrose in Q208 genotype as calculated by LSD post-hoc testing. The results relate to figure 3a. Letters indicate the presence of a significant difference between values within the same genotype. **Figure S5**. Screenshot of Tables presenting LSD post-hoc testing of cell wall component ratio in KQ228 genotype. The results relate to figure 3b. Screenshots derived from SPSS statistical package. Refer to Table 1 for numbers corresponding to tissue type. Abbreviations, TI: Top Internode; MI: Middle Internode; BI: Bottom Internode; 1st Visible Dewlap Leaf: L1; 5th Visible Dewlap Leaf: L5; R: Root. **Table S6**. Homogenous subsets of cell wall component ratio in KQ228 genotype as calculated by LSD post-hoc testing. The results relate to figure 3b. Letters indicate the presence of a significant difference between values within the same genotype. **Figure S6**. Screenshot of Tables presenting LSD post-hoc testing of cell wall component ratio in Q208 genotype. The results relate to figure 3b. Screenshots derived from SPSS statistical package. Refer to Table 1 for numbers corresponding to tissue type. Abbreviations, TI: Top Internode; MI: Middle Internode; BI: Bottom Internode; 1st Visible Dewlap Leaf: L1; 5th Visible Dewlap Leaf: L5; R: Root. **Table S7**. Homogenous subsets of cell wall component ratio in Q208 genotype as calculated by LSD post-hoc testing. The results relate to figure 3b. Letters indicate the presence of a significant difference between values within the same genotype. **Figure S7.** Screenshot of Tables presenting LSD post-hoc testing of non-cellulosic fractions in KQ228 genotype. The results relate to figure 3c. Screenshots derived from SPSS statistical package. Refer to Table 1 for numbers corresponding to tissue type. Abbreviations, TI: Top Internode; MI: Middle Internode; BI: Bottom Internode; 1st Visible Dewlap Leaf: L1; 5th Visible Dewlap Leaf: L5; R: Root. **Table S8.** Homogenous subsets of non-cellulosic fractions in KQ228 genotype as calculated by LSD post-hoc testing. The results relate to figure 3c. Letters indicate the presence of a significant difference between values within the same genotype. **Figure S8**. Screenshot of Tables presenting LSD post-hoc testing of non-cellulosic fractions in Q208 genotype. The results relate to figure 3c. Screenshots derived from SPSS statistical package. Refer to Table 1 for numbers corresponding to tissue type. Abbreviations, TI: Top Internode; MI: Middle Internode; BI: Bottom Internode; 1st Visible Dewlap Leaf: L1; 5th Visible Dewlap Leaf: L5; R: Root. **Table S9**. Homogenous subsets of non-cellulosic fractions in Q208 genotype. The results relate to figure 3c. Letters indicate the presence of a significant difference between values within the same genotype. **Figure S9**. Screenshot of Tables presenting LSD post-hoc testing of AIL/ASL lignin fractions in KQ228 genotype. The results relate to figure 3d. Screenshots derived from SPSS statistical package. Refer to Table 1 for numbers corresponding to tissue type. Abbreviations, TI: Top Internode; MI: Middle Internode; BI: Bottom Internode; 1st Visible Dewlap Leaf: L1; 5th Visible Dewlap Leaf: L5; R: Root. **Table S10**. Homogenous subsets of AIL/ASL lignin fractions in KQ228 genotype as calculated by LSD post-hoc testing. The results relate to figure 3d. Letters indicate the presence of a significant difference between values within the same genotype.**Figure S10.** Screenshot of Tables presenting LSD post-hoc testing of AIL/ASL lignin fractions in Q208 genotype. The results relate to figure 3d. Screenshots derived from SPSS statistical package. Refer to Table 1 for numbers corresponding to tissue type. Abbreviations, TI: Top Internode; MI: Middle Internode; BI: Bottom Internode; 1st Visible Dewlap Leaf: L1; 5th Visible Dewlap Leaf: L5; R: Root. **Table S11.** Homogenous subsets of AIL/ASL lignin fractions in Q208 genotype as calculated by LSD post-hoc testing. The results relate to figure 3d. Letters indicate the presence of a significant difference between values within the same genotype. **Figure S11**. Screenshot of Tables presenting LSD post-hoc testing of syringyl, guaiacyl and hydroxycinnamic lignin fractions in KQ228 genotype. The results relate to figure 4. Screenshots derived from SPSS statistical package. Refer to Table 1 for numbers corresponding to tissue type. Abbreviations, TI: Top Internode; MI: Middle Internode; BI: Bottom Internode; 1st Visible Dewlap Leaf: L1; 5th Visible Dewlap Leaf: L5; R: Root. **Table S12**. Homogenous subsets of syringyl, guaiacyl and hydroxycinnamic lignin fractions in KQ228 genotype as calculated by LSD post-hoc testing. The results relate to figure 4. Letters indicate the presence of a significant difference between values within the same genotype. **Figure S12**. Screenshot of Tables presenting LSD post-hoc testing of syringyl, guaiacyl and hydroxycinnamic lignin fractions in Q208 genotype. The results relate to figure 4. Screenshots derived from SPSS statistical package. Refer to Table 1 for numbers corresponding to tissue type. Abbreviations, TI: Top Internode; MI: Middle Internode; BI: Bottom Internode; 1st Visible Dewlap Leaf: L1; 5th Visible Dewlap Leaf: L5; R: Root. **Table S13.** Homogenous subsets of syringyl, guaiacyl and hydroxycinnamic lignin fractions in Q208 genotype as calculated by LSD post-hoc testing. The results relate to figure 4. Letters indicate the presence of a significant difference between values within the same genotype. **Figure S13**. Screenshot of Table presenting LSD post-hoc testing of syringyl and guaiacyl lignin ratio in KQ228 genotype. The results relate to table 2. Screenshots derived from SPSS statistical package. Refer to Table 1 for numbers corresponding to tissue type. Abbreviations, TI: Top Internode; MI: Middle Internode; BI: Bottom Internode; 1st Visible Dewlap Leaf: L1; 5th Visible Dewlap Leaf: L5; R: Root. **Table S14.** Homogenous subsets of syringyl and guaiacyl lignin in KQ228 genotype as calculated by LSD post-hoc testing. The results relate to table 2. Letters indicate the presence of a significant difference between values within the same genotype. **Figure S14.** Screenshot of Table presenting LSD post-hoc testing of syringyl and guaiacyl lignin ratio in Q208 genotype. The results relate to table 2. Screenshots derived from SPSS statistical package. Refer to Table 1 for numbers corresponding to tissue type. Abbreviations, TI: Top Internode; MI: Middle Internode; BI: Bottom Internode; 1st Visible Dewlap Leaf: L1; 5th Visible Dewlap Leaf: L5; R: Root. **Table S15**. Homogenous subsets of syringyl and guaiacyl lignin in Q208 genotype as calculated by LSD post-hoc testing. The results relate to table 2. Letters indicate the presence of a significant difference between values within the same genotype. **Figure S15.** Screenshot of Table presenting LSD post-hoc testing of xylan and glucan release in the KQ228 genotype. The results relate to figure 5 in main document. Screenshots derived from SPSS statistical package. Refer to Table 1 for numbers corresponding to tissue type. Abbreviations, TI: Top Internode; MI: Middle Internode; BI: Bottom Internode; 1st Visible Dewlap Leaf: L1; 5th Visible Dewlap Leaf: L5; R: Root. **Table S16. **Homogenous subsets of xylan and glucan release in KQ228 genotype as calculated by LSD post-hoc testing. The results relate to figure 5 in main document. Letters indicate the presence of significant difference between values within the same genotype. **Figure S16.** Screenshot of Table presenting LSD post-hoc testing of xylan and glucan release in Q208 genotype. The results relate to figure 5 in main document. Screenshots derived from SPSS statistical package. Refer to Table 1 for numbers corresponding to tissue type. Abbreviations, TI: Top Internode; MI: Middle Internode; BI: Bottom Internode; 1st Visible Dewlap Leaf: L1; 5th Visible Dewlap Leaf: L5; R: Root. **Table S17.** Homogenous subsets of xylan and glucan release in Q208 genotype as calculated by LSD post-hoc testing. The results relate to figure 5 in main document. Letters indicate the presence of significant difference between values within the same genotype.

## Data Availability

The datasets supporting the conclusions of this article are included in the article and its Additional file 1.

## Abbreviations

C: Carbon
S: Syringyl lignin
G: Guaiacyl lignin
HC: Hydroxycinnamic acids
S/G ratio: Syringyl–guaiacyl ratio
SuSy: Sucrose synthase
SPS: Sucrose phosphate synthase
BI: Bottom internode range
MI: Middle internode range
TI: Top Internode range
L1: First visible dewlap leaf
L5: Fifth visible dewlap leaf
R: Root
DW: Dry weight
AIR: Alcohol insoluble residue (extractives free)
