# Supplementary material for: Variation in sugarcane biomass composition and enzymatic saccharification of leaves, internodes and roots
Source: Biotechnol Biofuels. 2020 Dec 9;13:201. doi: 10.1186/s13068-020-01837-2 (PMC7724889; doi:10.1186/s13068-020-01837-2)
Supplement: Supplementary file 1 — Additional file 1: Table S1. Numerical Identifier for LSD post hoc test results. Figure S1. Screenshot of Tables presenting LSD post-hoc testing of major biomass fractions in KQ228 genotype. The results relate to figure 2. Screenshots derived from SPSS statistical package. Refer to Table 1 for numbers corresponding to tissue type. Abbreviations, TI: Top Internode; MI: Middle Internode; BI: Bottom Internode; 1st Visible Dewlap Leaf: L1; 5th Visible Dewlap Leaf: L5; R: Root. Table S2. Homogenous subsets of major biomass fractions in KQ228 genotype as calculated by LSD post-hoc testing. The results relate to figure 2. Letters indicate the presence of a significant difference between values within the same genotype. Figure S2. Screenshot of Tables presenting LSD post-hoc testing of major biomass fractions in Q208 genotype. The results relate to figure 2. Screenshots derived from SPSS statistical package. Refer to Table 1 for numbers corresponding to tissue type. Abbreviations, TI: Top Internode; MI: Middle Internode; BI: Bottom Internode; 1st Visible Dewlap Leaf: L1; 5th Visible Dewlap Leaf: L5; R: Root. Table S3. Homogenous subsets of major biomass fractions in Q208 genotype as calculated by LSD post-hoc testing. The results relate to figure 2. Letters indicate the presence of a significant difference between values within the same genotype. Figure S3. Screenshot of Tables presenting LSD post-hoc testing of soluble sugar fractions, fructose, glucose and sucrose in KQ228 genotype. The results relate to figure 3a. Screenshots derived from SPSS statistical package. Refer to Table 1 for numbers corresponding to tissue type. Abbreviations, TI: Top Internode; MI: Middle Internode; BI: Bottom Internode; 1st Visible Dewlap Leaf: L1; 5th Visible Dewlap Leaf: L5; R: Root. Table S4. Homogenous subsets of soluble sugar fractions, fructose, glucose and sucrose in KQ228 genotype as calculated by LSD post-hoc testing. The results relate to figure 3a. Letters indicate the presence of [file 13068_2020_1837_MOESM1_ESM.pdf]

# Supplementary Files 1

## Title: *"Variation in sugarcane biomass composition and enzymatic saccharification of leaves, internodes and roots"*

Authors: Patrick J Mason [1], Agnelo Furtado [1], Annelie Marquardt [2,3], Katrina Hodgson-Kratky [1], Nam V Hoang[1,4], Frederik C Botha [3], Gabriella Papa [5,6], Jenny C. Mortimer [6], Blake Simmons [6], Robert J Henry [1].

**Table 1. Numerical Identifier for LSD post hoc test results.**

| Tissue/Organ Type | Numerical Identifier |
|---|---|
| Bottom Internode (BI) | 1 |
| Middle Internode (MI) | 2 |
| Top Internode (TI) | 3 |
| Root (R) | 4 |
| 1st Visible Dewlap Leaf (L1) | 5 |
| 5th Visible Dewlap Leaf (L5) | 6 |

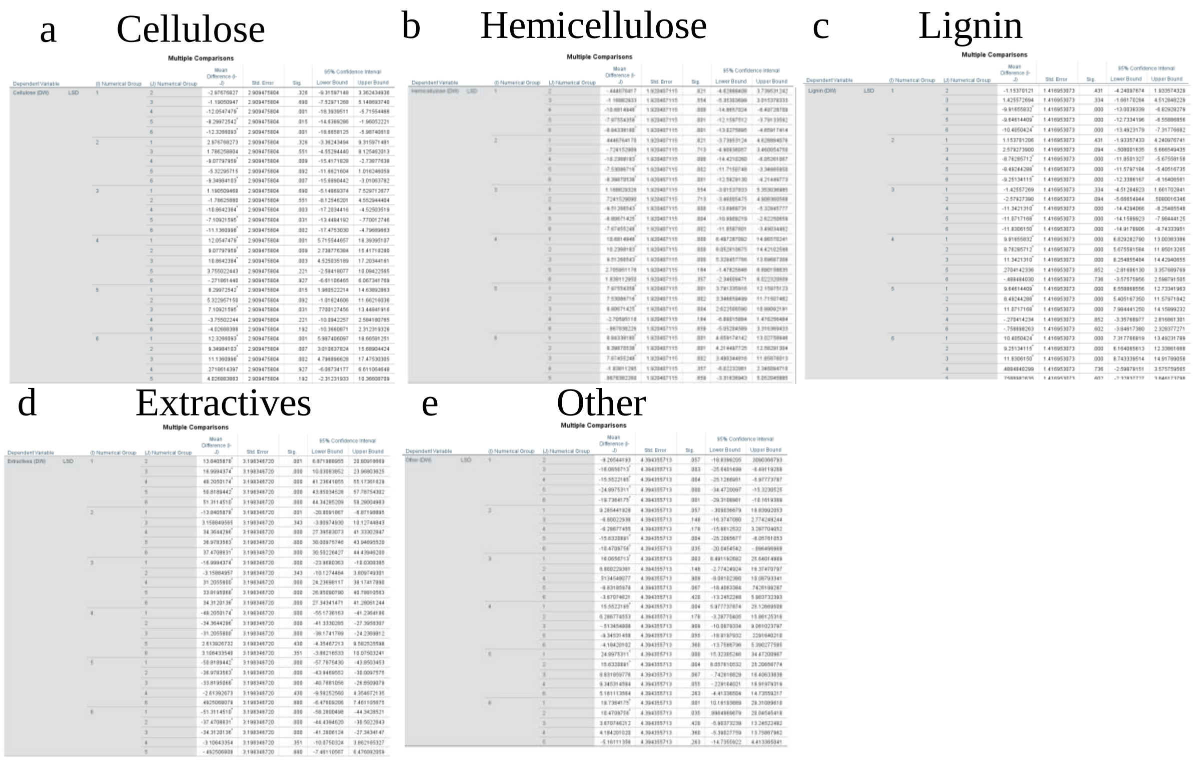


**Figure 1. Screenshot of Tables presenting LSD post-hoc testing of major biomass fractions in KQ228 genotype.** The results relate to figure 2 in main document. Screenshots derived from SPSS statistical package. Refer to Table 1 for numbers corresponding to tissue type. Abbreviations, TI: Top Internode; MI: Middle Internode; BI: Bottom Internode; 1$^{st}$ Visible Dewlap Leaf: L1; 5$^{th}$ Visible Dewlap Leaf: L5; R: Root.

**Table 2. Homogenous subsets of major biomass fractions in KQ228 genotype as calculated by LSD post-hoc testing.** The results relate to figure 1 in main document. Letters indicate the presence of significant difference between values within the same genotype.

| Tissue Type | Homogenous Subsets | | | | |
|---|---|---|---|---|---|
| | Cellulose | Hemicellulose | Lignin | Extractives | Other |
| **BI** | a | a | a | a | a |
| **MI** | a | a | a | b | ab |
| **TI** | ab | a | a | b | bc |
| **R** | c | b | b | c | bc |
| **L1** | bc | b | b | c | c |
| **L5** | b | b | b | c | c |

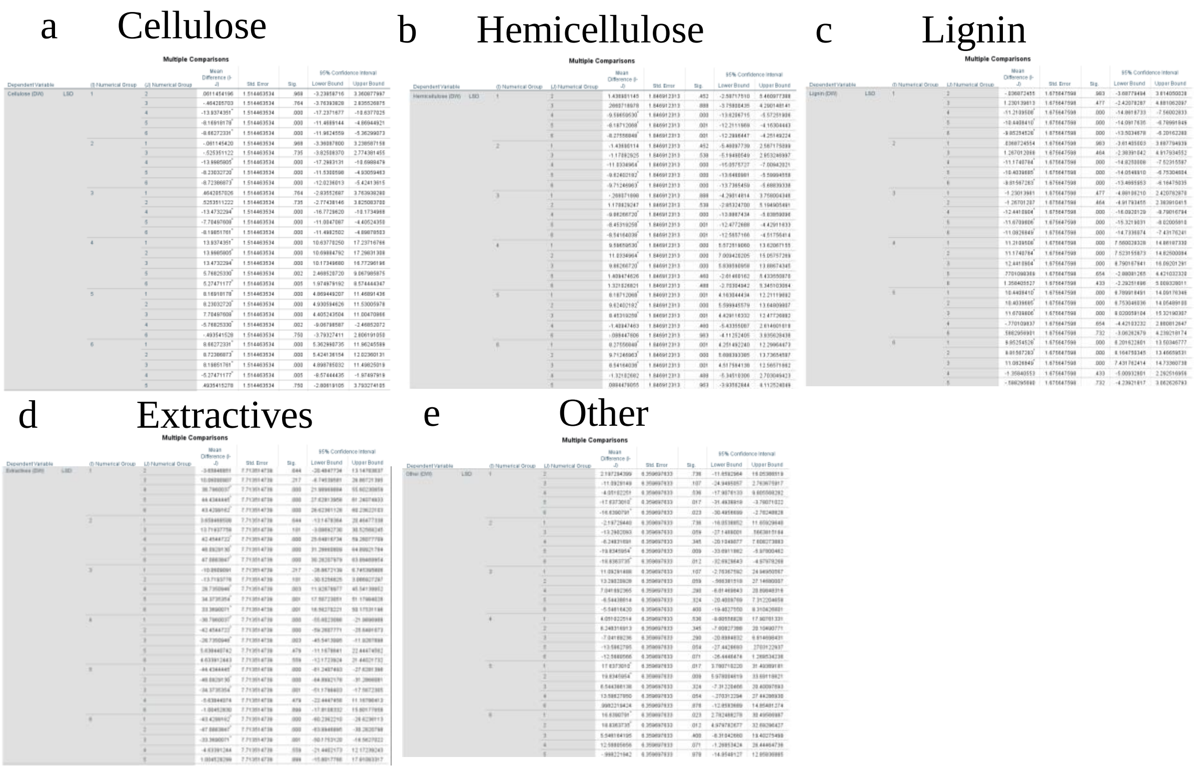


**Figure 2. Screenshot of Tables presenting LSD post-hoc testing of major biomass fractions in Q208 genotype.** Screenshots derived from SPSS statistical package. Refer to Table 1 for numbers corresponding to tissue type. Abbreviations, TI: Top Internode; MI: Middle Internode; BI: Bottom Internode; 1$^{st}$ Visible Dewlap Leaf: L1; 5$^{th}$ Visible Dewlap Leaf: L5; R: Root.

**Table 3. Homogenous subsets of major biomass fractions in Q208 genotype as calculated by LSD post-hoc testing.** The results relate to figure 2 in main document. Letters indicate the presence of significant difference between values within the same genotype.

| Tissue Type | Homogenous Subsets | | | | |
|---|---|---|---|---|---|
| | Cellulose | Hemicellulose | Lignin | Extractives | Other |
| **BI** | a | a | a | a | a |
| **MI** | a | a | a | a | a |
| **TI** | a | a | a | a | ab |
| **R** | c | b | b | b | ab |
| **L1** | b | b | b | b | b |
| **L5** | b | b | b | b | b |

a Sucrose

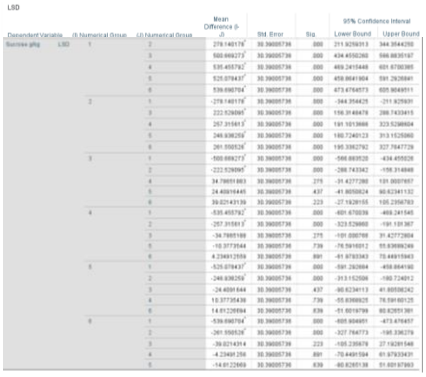


b Glucose

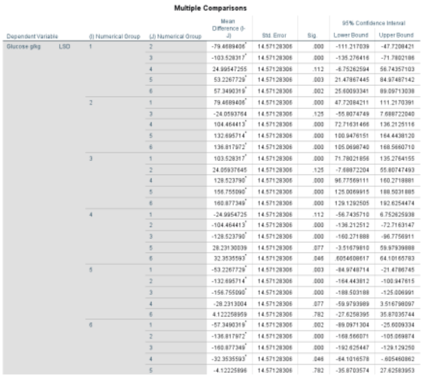


c Fructose

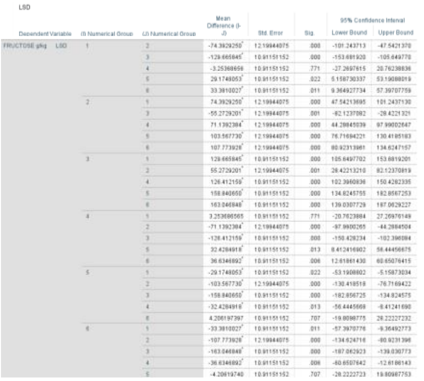


**Figure 3. Screenshot of Tables presenting LSD post-hoc testing of soluble sugar fractions, fructose, glucose and sucrose in KQ228 genotype.** The results relate to figure 3a in main document. Screenshots derived from SPSS statistical package. Refer to Table 1 for numbers corresponding to tissue type. Abbreviations, TI: Top Internode; MI: Middle Internode; BI: Bottom Internode; 1st Visible Dewlap Leaf: L1; 5th Visible Dewlap Leaf: L5; R: Root.

**Table 4. Homogenous subsets of soluble sugar fractions, fructose, glucose and sucrose in KQ228 genotype as calculated by LSD post-hoc testing.** The results relate to figure 3a in main document. Letters indicate the presence of significant difference between values within the same genotype.

| Tissue Type | Homogenous Subsets | | |
|---|---|---|---|
| | Sucrose | Glucose | Fructose |
| **BI** | a | b | c |
| **MI** | b | a | b |
| **TI** | c | a | a |
| **R** | c | bc | c |
| **L1** | c | c | d |
| **L5** | c | c | d |

a Sucrose

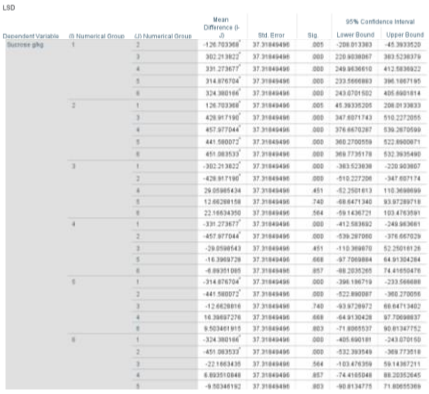

b Glucose

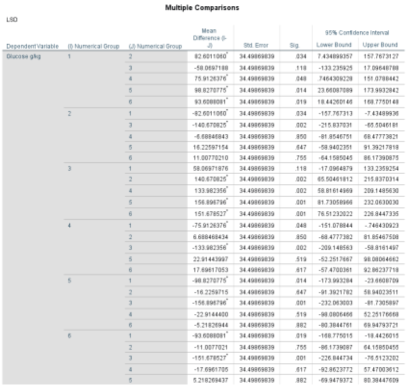

c Fructose

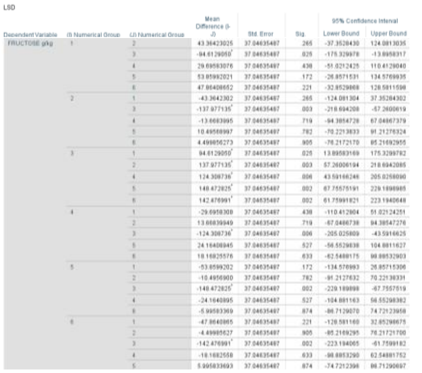

**Figure 4. Screenshot of Tables presenting LSD post-hoc testing of soluble sugar fractions, fructose, glucose and sucrose in Q208 genotype.** The results relate to figure 3a in main document. Screenshots derived from SPSS statistical package. Refer to Table 1 for numbers corresponding to tissue type. Abbreviations, TI: Top Internode; MI: Middle Internode; BI: Bottom Internode; 1st Visible Dewlap Leaf: L1; 5th Visible Dewlap Leaf: L5; R: Root.

**Table 5. Homogenous subsets of soluble sugar fractions, fructose, glucose and sucrose in Q208 genotype as calculated by LSD post-hoc testing.** The results relate to figure 3a in main document. Letters indicate the presence of significant difference between values within the same genotype.

| Tissue Type | Homogenous Subsets | | |
|---|---|---|---|
| | Sucrose | Glucose | Fructose |
| BI | b | a | a |
| MI | a | b | a |
| TI | c | a | b |
| R | c | b | a |
| L1 | c | b | a |
| L5 | c | b | a |

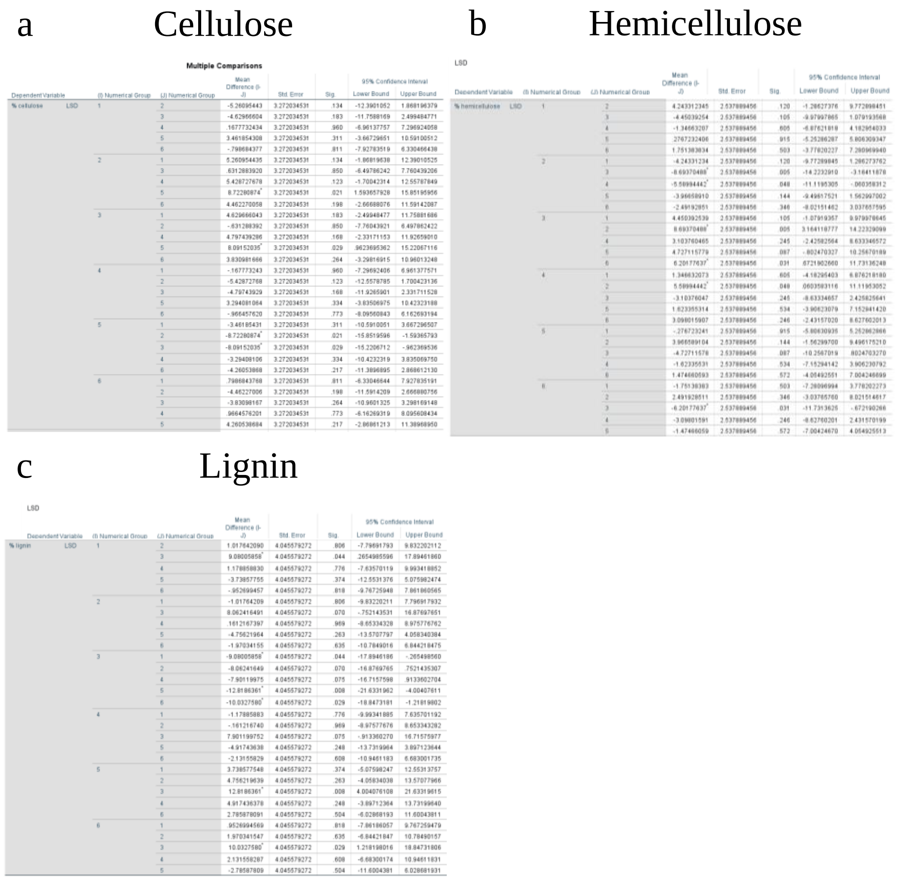


**Figure 5. Screenshot of Tables presenting LSD post-hoc testing of cell wall component ratio in KQ228 genotype.** The results relate to figure 3b in main document. Screenshots derived from SPSS statistical package. Refer to Table 1 for numbers corresponding to tissue type. Abbreviations, TI: Top Internode; MI: Middle Internode; BI: Bottom Internode; 1$^{st}$ Visible Dewlap Leaf: L1; 5$^{th}$ Visible Dewlap Leaf: L5; R: Root.

**Table 6. Homogenous subsets of cell wall component ratio in KQ228 genotype as calculated by LSD post-hoc testing.** The results relate to figure 3b in main document. Letters indicate the presence of significant difference between values within the same genotype.

| Tissue Type | Homogenous Subsets | | |
|---|---|---|---|
| | Cellulose | Hemicellulose | Lignin |
| **BI** | ab | ab | ab |
| **MI** | a | b | ab |
| **TI** | a | a | a |
| **R** | ab | ab | ab |
| **L1** | b | ab | b |
| **L5** | ab | b | b |

a Cellulose

**Multiple Comparisons**

| Dependent Variable | | (I) Numerical Group | (J) Numerical Group | Mean Difference (I-J) | Std. Error | Sig. | 95% Confidence Interval Lower Bound | Upper Bound |
|---|---|---|---|---|---|---|---|---|
| % cellulose | LSD | 1 | 2 | -2.95623017 | 2.908196217 | .329 | -9.29264540 | 3.380185061 |
| | | | 3 | -5.58439024 | 2.908196217 | .079 | -11.9208055 | .7520249904 |
| | | | 4 | -1.30109677 | 2.908196217 | .663 | -7.63751200 | 5.035318460 |
| | | | 5 | 4.291691019 | 2.908196217 | .166 | -2.04472421 | 10.62810625 |
| | | | 6 | 3.371890938 | 2.908196217 | .269 | -2.96452429 | 9.708306167 |
| | | 2 | 1 | 2.956230168 | 2.908196217 | .329 | -3.38018506 | 9.292645397 |
| | | | 3 | -2.62816007 | 2.908196217 | .384 | -8.96457530 | 3.708255159 |
| | | | 4 | 1.655133399 | 2.908196217 | .580 | -4.68128183 | 7.991548628 |
| | | | 5 | 7.24792119* | 2.908196217 | .028 | .9115059579 | 13.58433642 |
| | | | 6 | 6.328121106 | 2.908196217 | .050 | -.008294123 | 12.66453633 |
| | | 3 | 1 | 5.584390239 | 2.908196217 | .079 | -.752024990 | 11.92080547 |
| | | | 2 | 2.628160070 | 2.908196217 | .384 | -3.70825516 | 8.964575299 |
| | | | 4 | 4.283293469 | 2.908196217 | .167 | -2.05312176 | 10.61970870 |
| | | | 5 | 9.87608126* | 2.908196217 | .005 | 3.539666028 | 16.21249649 |
| | | | 6 | 8.95628118* | 2.908196217 | .010 | 2.619865947 | 15.29269641 |
| | | 4 | 1 | 1.301096769 | 2.908196217 | .663 | -5.03531846 | 7.637511998 |
| | | | 2 | -1.65513340 | 2.908196217 | .580 | -7.99154863 | 4.681281830 |
| | | | 3 | -4.28329347 | 2.908196217 | .167 | -10.6197087 | 2.053121760 |
| | | | 5 | 5.592787788 | 2.908196217 | .079 | -.743627441 | 11.92920302 |
| | | | 6 | 4.672987707 | 2.908196217 | .134 | -1.66342752 | 11.00940294 |
| | | 5 | 1 | -4.29169102 | 2.908196217 | .166 | -10.6281062 | 2.044724210 |
| | | | 2 | -7.24792119* | 2.908196217 | .028 | -13.5843364 | -.911505958 |
| | | | 3 | -9.87608126* | 2.908196217 | .005 | -16.2124965 | -3.53966603 |
| | | | 4 | -5.59278779 | 2.908196217 | .079 | -11.9292030 | .7436274411 |
| | | | 6 | -.919800081 | 2.908196217 | .757 | -7.25621531 | 5.416615148 |
| | | 6 | 1 | -3.37189094 | 2.908196217 | .269 | -9.70830617 | 2.964524291 |
| | | | 2 | -6.32812111 | 2.908196217 | .050 | -12.6645363 | .0082941231 |
| | | | 3 | -8.95628118* | 2.908196217 | .010 | -15.2926964 | -2.61986595 |
| | | | 4 | -4.67298771 | 2.908196217 | .134 | -11.0094029 | 1.663427522 |
| | | | 5 | .9198000810 | 2.908196217 | .757 | -5.41661515 | 7.256215310 |

b Hemicellulose

LSD

| Dependent Variable | | (I) Numerical Group | (J) Numerical Group | Mean Difference (I-J) | Std. Error | Sig. | 95% Confidence Interval Lower Bound | Upper Bound |
|---|---|---|---|---|---|---|---|---|
| hemicellulose | LSD | 1 | 2 | 5.196978116 | 3.392335231 | .151 | -2.19428541 | 12.58824164 |
| | | | 3 | .5761750485 | 3.392335231 | .868 | -6.81508848 | 7.967438573 |
| | | | 4 | 5.955217392 | 3.392335231 | .105 | -1.43604613 | 13.34648092 |
| | | | 5 | 3.603679185 | 3.392335231 | .309 | -3.78758434 | 10.99494271 |
| | | | 6 | 3.413942566 | 3.392335231 | .334 | -3.97732096 | 10.80520609 |
| | | 2 | 1 | -5.19697812 | 3.392335231 | .151 | -12.5882416 | 2.194285409 |
| | | | 3 | -4.62080307 | 3.392335231 | .198 | -12.0120666 | 2.770460457 |
| | | | 4 | .7582392761 | 3.392335231 | .827 | -6.63302425 | 8.149502801 |
| | | | 5 | -1.59329893 | 3.392335231 | .647 | -8.98456246 | 5.797964594 |
| | | | 6 | -1.78303555 | 3.392335231 | .609 | -9.17429907 | 5.608227975 |
| | | 3 | 1 | -.576175049 | 3.392335231 | .868 | -7.96743857 | 6.815088476 |
| | | | 2 | 4.620803068 | 3.392335231 | .198 | -2.77046046 | 12.01206659 |
| | | | 4 | 5.379042344 | 3.392335231 | .139 | -2.01222118 | 12.77030587 |
| | | | 5 | 3.027504137 | 3.392335231 | .390 | -4.36375939 | 10.41876766 |
| | | | 6 | 2.837767518 | 3.392335231 | .419 | -4.55349601 | 10.22903104 |
| | | 4 | 1 | -5.95521739 | 3.392335231 | .105 | -13.3464809 | 1.436046132 |
| | | | 2 | -.758239276 | 3.392335231 | .827 | -8.14950280 | 6.633024249 |
| | | | 3 | -5.37904234 | 3.392335231 | .139 | -12.7703059 | 2.012221181 |
| | | | 5 | -2.35153821 | 3.392335231 | .501 | -9.74280173 | 5.039725318 |
| | | | 6 | -2.54127483 | 3.392335231 | .468 | -9.93253835 | 4.849988699 |
| | | 5 | 1 | -3.60367919 | 3.392335231 | .309 | -10.9949427 | 3.787584339 |
| | | | 2 | 1.593298931 | 3.392335231 | .647 | -5.79796459 | 8.984562455 |
| | | | 3 | -3.02750414 | 3.392335231 | .390 | -10.4187677 | 4.363759388 |
| | | | 4 | 2.351538207 | 3.392335231 | .501 | -5.03972532 | 9.742801732 |
| | | | 6 | -.189736619 | 3.392335231 | .956 | -7.58100014 | 7.201526906 |
| | | 6 | 1 | -3.41394257 | 3.392335231 | .334 | -10.8052061 | 3.977320958 |
| | | | 2 | 1.783035550 | 3.392335231 | .609 | -5.60822797 | 9.174299074 |
| | | | 3 | -2.83776752 | 3.392335231 | .419 | -10.2290310 | 4.553496007 |
| | | | 4 | 2.541274826 | 3.392335231 | .468 | -4.84998870 | 9.932538350 |
| | | | 5 | .1897366190 | 3.392335231 | .956 | -7.20152691 | 7.581000144 |

c Lignin

LSD

| Dependent Variable | | (I) Numerical Group | (J) Numerical Group | Mean Difference (I-J) | Std. Error | Sig. | 95% Confidence Interval Lower Bound | Upper Bound |
|---|---|---|---|---|---|---|---|---|
| % lignin | LSD | 1 | 2 | -2.24074795 | 3.357687811 | .517 | -9.55652123 | 5.075025333 |
| | | | 3 | 5.008215190 | 3.357687811 | .162 | -2.30755809 | 12.32398847 |
| | | | 4 | -4.65412062 | 3.357687811 | .191 | -11.9698939 | 2.661652658 |
| | | | 5 | -7.89537020* | 3.357687811 | .037 | -15.2111435 | -.579596923 |
| | | | 6 | -6.78583350 | 3.357687811 | .066 | -14.1016068 | .5299397767 |
| | | 2 | 1 | 2.240747948 | 3.357687811 | .517 | -5.07502533 | 9.556521228 |
| | | | 3 | 7.248963138 | 3.357687811 | .052 | -.066810143 | 14.56473642 |
| | | | 4 | -2.41337268 | 3.357687811 | .486 | -9.72914596 | 4.902400605 |
| | | | 5 | -5.65462226 | 3.357687811 | .118 | -12.9703955 | 1.661151024 |
| | | | 6 | -4.54508556 | 3.357687811 | .201 | -11.8608588 | 2.770687724 |
| | | 3 | 1 | -5.00821519 | 3.357687811 | .162 | -12.3239885 | 2.307558090 |
| | | | 2 | -7.24896314 | 3.357687811 | .052 | -14.5647364 | .0668101428 |
| | | | 4 | -9.66233581* | 3.357687811 | .014 | -16.9781091 | -2.34656253 |
| | | | 5 | -12.9035854* | 3.357687811 | .002 | -20.2193587 | -5.58781211 |
| | | | 6 | -11.7940487* | 3.357687811 | .004 | -19.1098220 | -4.47827541 |
| | | 4 | 1 | 4.654120623 | 3.357687811 | .191 | -2.66165266 | 11.96989390 |
| | | | 2 | 2.413372675 | 3.357687811 | .486 | -4.90240061 | 9.729145956 |
| | | | 3 | 9.66233581* | 3.357687811 | .014 | 2.346562532 | 16.97810909 |
| | | | 5 | -3.24124958 | 3.357687811 | .353 | -10.5570229 | 4.074523700 |
| | | | 6 | -2.13171288 | 3.357687811 | .537 | -9.44748616 | 5.184060399 |
| | | 5 | 1 | 7.89537020* | 3.357687811 | .037 | .5795969232 | 15.21114348 |
| | | | 2 | 5.654622256 | 3.357687811 | .118 | -1.66115102 | 12.97039554 |
| | | | 3 | 12.9035854* | 3.357687811 | .002 | 5.587812113 | 20.21935867 |
| | | | 4 | 3.241249581 | 3.357687811 | .353 | -4.07452370 | 10.55702286 |
| | | | 6 | 1.109536700 | 3.357687811 | .747 | -6.20623658 | 8.425309980 |
| | | 6 | 1 | 6.785833504 | 3.357687811 | .066 | -.529939777 | 14.10160678 |
| | | | 2 | 4.545085556 | 3.357687811 | .201 | -2.77068772 | 11.86085884 |
| | | | 3 | 11.7940487* | 3.357687811 | .004 | 4.478275413 | 19.10982197 |
| | | | 4 | 2.131712881 | 3.357687811 | .537 | -5.18406040 | 9.447486162 |
| | | | 5 | -1.10953670 | 3.357687811 | .747 | -8.42530998 | 6.206236581 |

**Figure 6. Screenshot of Tables presenting LSD post-hoc testing of cell wall component ratio in Q208 genotype.** The results relate to figure 3b in main document. Screenshots derived from SPSS statistical package. Refer to Table 1 for numbers corresponding to tissue type. Abbreviations, TI: Top Internode; MI: Middle Internode; BI: Bottom Internode; 1$^{st}$ Visible Dewlap Leaf: L1; 5$^{th}$ Visible Dewlap Leaf: L5; R: Root.

**Table 7. Homogenous subsets of cell wall component ratio in Q208 genotype as calculated by LSD post-hoc testing.** The results relate to figure 3b in main document. Letters indicate the presence of significant difference between values within the same genotype.

| Tissue Type | Homogenous Subsets | | |
|---|---|---|---|
| | Cellulose | Hemicellulose | Lignin |
| BI | abc | a | ab |
| MI | ab | a | ab |
| TI | a | a | a |
| R | abc | a | b |
| L1 | c | a | b |
| L5 | bc | a | b |

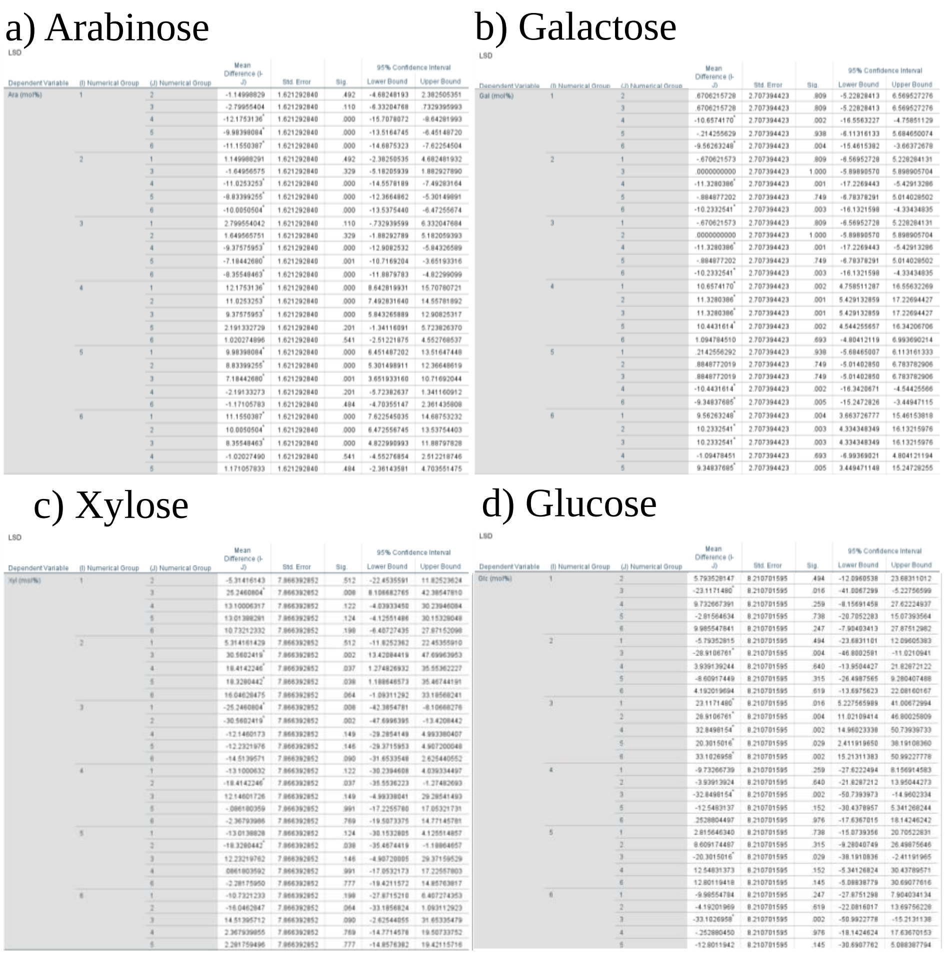


**Figure 7. Screenshot of Tables presenting LSD post-hoc testing of hemicellulose fractions in KQ228 genotype.** The results relate to figure 3c in main document. Screenshots derived from SPSS statistical package. Refer to Table 1 for numbers corresponding to tissue type. Abbreviations, TI: Top Internode; MI: Middle Internode; BI: Bottom Internode; 1$^{st}$ Visible Dewlap Leaf: L1; 5$^{th}$ Visible Dewlap Leaf: L5; R: Root.

**Table 8. Homogenous subsets of hemicellulose fractions in KQ228 genotype as calculated by LSD post-hoc testing.** The results relate to figure 3c in main document. Letters indicate the presence of significant difference between values within the same genotype.

| Tissue Type | Homogenous Subsets | | | |
|---|---|---|---|---|
| | Arabinose | Galactose | Glucose | Xylose |
| BI | a | a | a | a |
| MI | a | a | a | a |
| TI | a | a | b | b |
| R | b | b | a | ab |
| L1 | b | a | a | ab |
| L5 | b | b | a | ab |

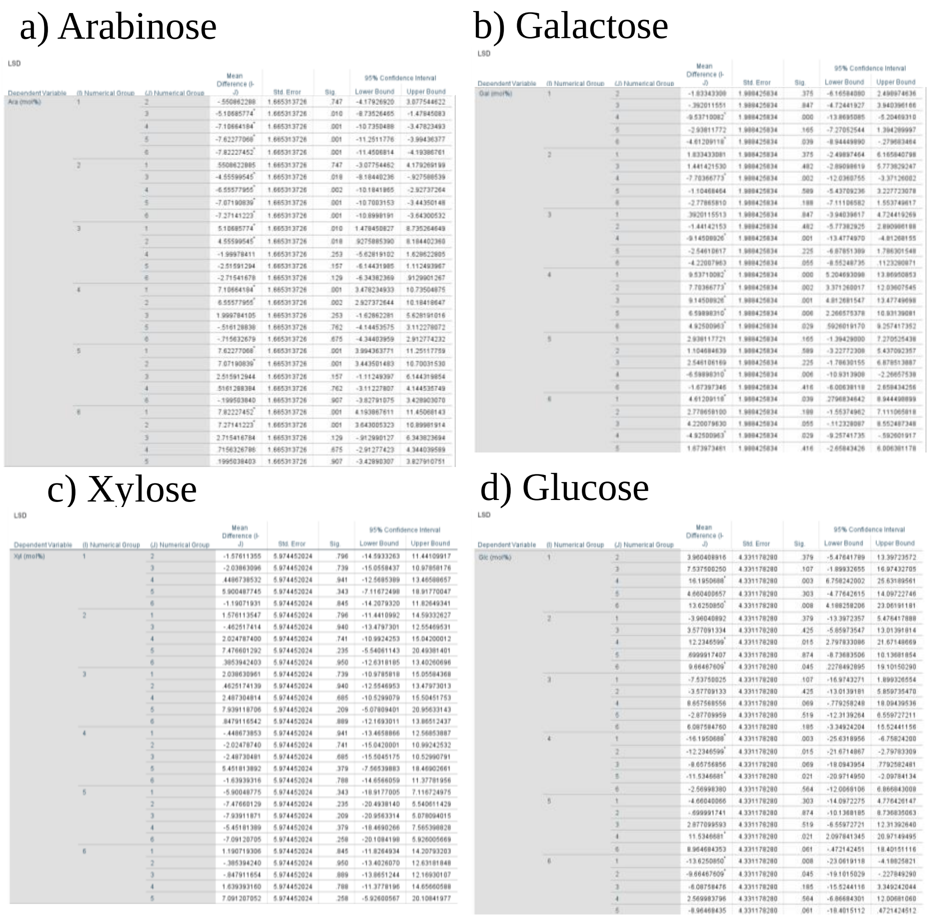


**Figure 8. Screenshot of Tables presenting LSD post-hoc testing of hemicellulose fractions in Q208 genotype.** The results relate to figure 3c in main document. Screenshots derived from SPSS statistical package. Refer to Table 1 for numbers corresponding to tissue type. Abbreviations, TI: Top Internode; MI: Middle Internode; BI: Bottom Internode; 1st Visible Dewlap Leaf: L1; 5th Visible Dewlap Leaf: L5; R: Root.

**Table 9. Homogenous subsets of hemicellulose fractions in Q208 genotype.** The results relate to figure 3c in main document. Letters indicate the presence of significant difference between values within the same genotype.

| Tissue Type | Homogenous Subsets | | | |
|---|---|---|---|---|
| | Arabinose | Galactose | Glucose | Xylose |
| **BI** | a | a | a | a |
| **MI** | a | a | ab | a |
| **TI** | b | a | abc | a |
| **R** | b | b | c | a |
| **L1** | b | a | ab | a |
| **L5** | b | a | b | a |

a) AIL

LSD

| Dependent Variable | | (I) Numerical Group | (J) Numerical Group | Mean Difference (I-J) | Std. Error | Sig. | 95% Confidence Interval | |
|---|---|---|---|---|---|---|---|---|
| | | | | | | | Lower Bound | Upper Bound |
| AIL% | LSD | 1 | 2 | 3.051808889 | 1.672321868 | .093 | -.591867442 | 6.695485240 |
| | | | 3 | 8.35721959* | 1.672321868 | .000 | 4.713543246 | 12.00089593 |
| | | | 4 | -1.35917429 | 1.672321868 | .432 | -5.00285963 | 2.284502050 |
| | | | 5 | 2.321137089 | 1.672321868 | .190 | -1.32253925 | 5.964813430 |
| | | | 6 | 1.660483768 | 1.672321868 | .340 | -1.98319257 | 5.304160109 |
| | | 2 | 1 | -3.05180890 | 1.672321868 | .093 | -6.69548524 | .5918674416 |
| | | | 3 | 5.30541069* | 1.672321868 | .008 | 1.661734347 | 8.949087029 |
| | | | 4 | -4.41098319* | 1.672321868 | .022 | -8.05465953 | -.767306850 |
| | | | 5 | -.730671811 | 1.672321868 | .670 | -4.37434815 | 2.913004530 |
| | | | 6 | -1.39132513 | 1.672321868 | .422 | -5.03500147 | 2.252351209 |
| | | 3 | 1 | -8.35721959* | 1.672321868 | .000 | -12.0008959 | -4.71354325 |
| | | | 2 | -5.30541069* | 1.672321868 | .008 | -8.94908703 | -1.66173435 |
| | | | 4 | -9.71639388* | 1.672321868 | .000 | -13.3600702 | -6.07271754 |
| | | | 5 | -6.03608250* | 1.672321868 | .004 | -9.67975884 | -2.39240616 |
| | | | 6 | -6.69673582* | 1.672321868 | .002 | -10.3404122 | -3.05305948 |
| | | 4 | 1 | 1.359174291 | 1.672321868 | .432 | -2.28450205 | 5.002850632 |
| | | | 2 | 4.41098319* | 1.672321868 | .022 | .7673068497 | 8.054659532 |
| | | | 3 | 9.71639388* | 1.672321868 | .000 | 6.072717537 | 13.36007022 |
| | | | 5 | 3.68031138* | 1.672321868 | .048 | .0366350392 | 7.323987721 |
| | | | 6 | 3.019658059 | 1.672321868 | .096 | -.624018282 | 6.663334400 |
| | | 5 | 1 | -2.32113709 | 1.672321868 | .190 | -5.96481343 | 1.322539252 |
| | | | 2 | .7306718105 | 1.672321868 | .670 | -2.91300453 | 4.374348152 |
| | | | 3 | 6.03608250* | 1.672321868 | .004 | 2.392406157 | 9.679758839 |
| | | | 4 | -3.68031138* | 1.672321868 | .048 | -7.32398772 | -.036635039 |
| | | | 6 | -.660653321 | 1.672321868 | .700 | -4.30432966 | 2.983023020 |
| | | 6 | 1 | -1.66048377 | 1.672321868 | .340 | -5.30416011 | 1.983192573 |
| | | | 2 | 1.391325132 | 1.672321868 | .422 | -2.25235121 | 5.035001473 |
| | | | 3 | 6.69673582* | 1.672321868 | .002 | 3.053059478 | 10.34041216 |
| | | | 4 | -3.01965806 | 1.672321868 | .096 | -6.66333440 | .6240182820 |
| | | | 5 | .6606533212 | 1.672321868 | .700 | -2.98302302 | 4.304329662 |

c) ASL

Multiple Comparisons

| Dependent Variable | | (I) Numerical Group | (J) Numerical Group | Mean Difference (I-J) | Std. Error | Sig. | 95% Confidence Interval | |
|---|---|---|---|---|---|---|---|---|
| | | | | | | | Lower Bound | Upper Bound |
| ASL% | LSD | 1 | 2 | .4640000000 | .3375831047 | .194 | -.271530400 | 1.199530400 |
| | | | 3 | 1.63560000* | .3375831047 | .000 | .9000696004 | 2.371130400 |
| | | | 4 | .870000000* | .3375831047 | .024 | .1344696004 | 1.605530400 |
| | | | 5 | -1.82893333* | .3375831047 | .000 | -2.56446373 | -1.09340293 |
| | | | 6 | -1.92173333* | .3375831047 | .000 | -2.65726373 | -1.18620293 |
| | | 2 | 1 | -.464000000 | .3375831047 | .194 | -1.19953040 | .2715303996 |
| | | | 3 | 1.17160000* | .3375831047 | .005 | .4360696004 | 1.907130400 |
| | | | 4 | .4060000000 | .3375831047 | .252 | -.329530400 | 1.141530400 |
| | | | 5 | -2.29293333* | .3375831047 | .000 | -3.02846373 | -1.55740293 |
| | | | 6 | -2.38573333* | .3375831047 | .000 | -3.12126373 | -1.65020293 |
| | | 3 | 1 | -1.63560000* | .3375831047 | .000 | -2.37113040 | -.900069600 |
| | | | 2 | -1.17160000* | .3375831047 | .005 | -1.90713040 | -.436069600 |
| | | | 4 | -.765600000* | .3375831047 | .043 | -1.50113040 | -.030069600 |
| | | | 5 | -3.46453333* | .3375831047 | .000 | -4.20006373 | -2.72900293 |
| | | | 6 | -3.55733333* | .3375831047 | .000 | -4.29286373 | -2.82180293 |
| | | 4 | 1 | -.870000000* | .3375831047 | .024 | -1.60553040 | -.134469600 |
| | | | 2 | -.406000000 | .3375831047 | .252 | -1.14153040 | .3295303996 |
| | | | 3 | .765600000* | .3375831047 | .043 | .0300696004 | 1.501130400 |
| | | | 5 | -2.69893333* | .3375831047 | .000 | -3.43446373 | -1.96340293 |
| | | | 6 | -2.79173333* | .3375831047 | .000 | -3.52726373 | -2.05620293 |
| | | 5 | 1 | 1.82893333* | .3375831047 | .000 | 1.093402934 | 2.564463733 |
| | | | 2 | 2.29293333* | .3375831047 | .000 | 1.557402934 | 3.028463733 |
| | | | 3 | 3.46453333* | .3375831047 | .000 | 2.729002934 | 4.200063733 |
| | | | 4 | 2.69893333* | .3375831047 | .000 | 1.963402934 | 3.434463733 |
| | | | 6 | -.092800000 | .3375831047 | .788 | -.828330400 | .6427303996 |
| | | 6 | 1 | 1.92173333* | .3375831047 | .000 | 1.186202934 | 2.657263733 |
| | | | 2 | 2.38573333* | .3375831047 | .000 | 1.650202934 | 3.121263733 |
| | | | 3 | 3.55733333* | .3375831047 | .000 | 2.821802934 | 4.292863733 |
| | | | 4 | 2.79173333* | .3375831047 | .000 | 2.056202934 | 3.527263733 |
| | | | 5 | .0928000000 | .3375831047 | .788 | -.642730400 | .8283303996 |

**Figure 9. Screenshot of Tables presenting LSD post-hoc testing of AIL/ASL lignin fractions in KQ228 genotype.** The results relate to figure 3d in main document. Screenshots derived from SPSS statistical package. Refer to Table 1 for numbers corresponding to tissue type. Abbreviations, TI: Top Internode; MI: Middle Internode; BI: Bottom Internode; 1$^{st}$ Visible Dewlap Leaf: L1; 5$^{th}$ Visible Dewlap Leaf: L5; R: Root.

**Table 10. Homogenous subsets of AIL/ASL lignin fractions in KQ228 genotype as calculated by LSD post-hoc testing.** The results relate to figure 3d in main document. Letters indicate the presence of significant difference between values within the same genotype.

| Tissue Type | Homogenous Subsets | |
|---|---|---|
| | ASL | AIL |
| **BI** | a | ac |
| **MI** | ac | a |
| **TI** | b | b |
| **R** | ac | c |
| **L1** | d | ac |
| **L5** | d | ac |

a) AIL

LSD

| Dependent Variable | | (I) Numerical Group | (J) Numerical Group | Mean Difference (I-J) | Std. Error | Sig. | 95% Confidence Interval | |
|---|---|---|---|---|---|---|---|---|
| | | | | | | | Lower Bound | Upper Bound |
| AIL% | LSD | 1 | 2 | -1.83892820 | 2.872909186 | .534 | -8.09845959 | 4.420603195 |
| | | | 3 | 4.732655342 | 2.872909186 | .125 | -1.52687605 | 10.99218674 |
| | | | 4 | -8.78564598* | 2.872909186 | .010 | -15.0451774 | -2.52611458 |
| | | | 5 | -3.03702214 | 2.872909186 | .311 | -9.29655354 | 3.222509250 |
| | | | 6 | -2.89398360 | 2.872909186 | .334 | -9.15351499 | 3.365547794 |
| | | 2 | 1 | 1.838928198 | 2.872909186 | .534 | -4.42060320 | 8.098459591 |
| | | | 3 | 6.57158354* | 2.872909186 | .041 | .3120521468 | 12.83111493 |
| | | | 4 | -6.94671778* | 2.872909186 | .032 | -13.2062492 | -.687186387 |
| | | | 5 | -1.19809395 | 2.872909186 | .684 | -7.45762534 | 5.061437448 |
| | | | 6 | -1.05505540 | 2.872909186 | .720 | -7.31458679 | 5.204475992 |
| | | 3 | 1 | -4.73265534 | 2.872909186 | .125 | -10.9921867 | 1.526876051 |
| | | | 2 | -6.57158354* | 2.872909186 | .041 | -12.8311149 | -.312052147 |
| | | | 4 | -13.5183013* | 2.872909186 | .001 | -19.7778327 | -7.25876993 |
| | | | 5 | -7.76967749* | 2.872909186 | .019 | -14.0292089 | -1.51014609 |
| | | | 6 | -7.62663894* | 2.872909186 | .021 | -13.8861703 | -1.36710755 |
| | | 4 | 1 | 8.78564598* | 2.872909186 | .010 | 2.526114584 | 15.04517737 |
| | | | 2 | 6.94671778* | 2.872909186 | .032 | .6871863865 | 13.20624917 |
| | | | 3 | 13.5183013* | 2.872909186 | .001 | 7.258769926 | 19.77783271 |
| | | | 5 | 5.748623834 | 2.872909186 | .069 | -.510907559 | 12.00815523 |
| | | | 6 | 5.891662378 | 2.872909186 | .063 | -.367869015 | 12.15119377 |
| | | 5 | 1 | 3.037022143 | 2.872909186 | .311 | -3.22250925 | 9.296553536 |
| | | | 2 | 1.198093946 | 2.872909186 | .684 | -5.06143745 | 7.457625339 |
| | | | 3 | 7.76967749* | 2.872909186 | .019 | 1.510146092 | 14.02920888 |
| | | | 4 | -5.74862383 | 2.872909186 | .069 | -12.0081552 | .5109075591 |
| | | | 6 | .1430385444 | 2.872909186 | .961 | -6.11649285 | 6.402569938 |
| | | 6 | 1 | 2.893983599 | 2.872909186 | .334 | -3.36554779 | 9.153514992 |
| | | | 2 | 1.055055401 | 2.872909186 | .720 | -5.20447599 | 7.314586794 |
| | | | 3 | 7.62663894* | 2.872909186 | .021 | 1.367107548 | 13.88617033 |
| | | | 4 | -5.89166238 | 2.872909186 | .063 | -12.1511938 | .3678690147 |
| | | | 5 | -.143038544 | 2.872909186 | .961 | -6.40256994 | 6.116492849 |

c) ASL

Multiple Comparisons

| Dependent Variable | | (I) Numerical Group | (J) Numerical Group | Mean Difference (I-J) | Std. Error | Sig. | 95% Confidence Interval | |
|---|---|---|---|---|---|---|---|---|
| | | | | | | | Lower Bound | Upper Bound |
| ASL% | LSD | 1 | 2 | -.143066667 | .4681648269 | .765 | -1.16311020 | .8769768647 |
| | | | 3 | 1.79800000* | .4681648269 | .002 | .7779564687 | 2.818043531 |
| | | | 4 | .9976000000 | .4681648269 | .054 | -.022443531 | 2.017643531 |
| | | | 5 | -1.64720000* | .4681648269 | .004 | -2.66724353 | -.627156469 |
| | | | 6 | -1.20253333* | .4681648269 | .025 | -2.22257686 | -.182489802 |
| | | 2 | 1 | .1430666667 | .4681648269 | .765 | -.876976865 | 1.163110198 |
| | | | 3 | 1.94106667* | .4681648269 | .001 | .9210231353 | 2.961110198 |
| | | | 4 | 1.14066667* | .4681648269 | .031 | .1206231353 | 2.160710198 |
| | | | 5 | -1.50413333* | .4681648269 | .007 | -2.52417686 | -.484089802 |
| | | | 6 | -1.05946667* | .4681648269 | .043 | -2.07951020 | -.039423135 |
| | | 3 | 1 | -1.79800000* | .4681648269 | .002 | -2.81804353 | -.777956469 |
| | | | 2 | -1.94106667* | .4681648269 | .001 | -2.96111020 | -.921023135 |
| | | | 4 | -.800400000 | .4681648269 | .113 | -1.82044353 | .2196435313 |
| | | | 5 | -3.44520000* | .4681648269 | .000 | -4.46524353 | -2.42515647 |
| | | | 6 | -3.00053333* | .4681648269 | .000 | -4.02057686 | -1.98048980 |
| | | 4 | 1 | -.997600000 | .4681648269 | .054 | -2.01764353 | .0224435313 |
| | | | 2 | -1.14066667* | .4681648269 | .031 | -2.16071020 | -.120623135 |
| | | | 3 | .8004000000 | .4681648269 | .113 | -.219643531 | 1.820443531 |
| | | | 5 | -2.64480000* | .4681648269 | .000 | -3.66484353 | -1.62475647 |
| | | | 6 | -2.20013333* | .4681648269 | .001 | -3.22017686 | -1.18008980 |
| | | 5 | 1 | 1.64720000* | .4681648269 | .004 | .6271564687 | 2.667243531 |
| | | | 2 | 1.50413333* | .4681648269 | .007 | .4840898020 | 2.524176865 |
| | | | 3 | 3.44520000* | .4681648269 | .000 | 2.425156469 | 4.465243531 |
| | | | 4 | 2.64480000* | .4681648269 | .000 | 1.624756469 | 3.664843531 |
| | | | 6 | .4446666667 | .4681648269 | .361 | -.575376865 | 1.464710198 |
| | | 6 | 1 | 1.20253333* | .4681648269 | .025 | .1824898020 | 2.222576865 |
| | | | 2 | 1.05946667* | .4681648269 | .043 | .0394231353 | 2.079510198 |
| | | | 3 | 3.00053333* | .4681648269 | .000 | 1.980489802 | 4.020576865 |
| | | | 4 | 2.20013333* | .4681648269 | .001 | 1.180089802 | 3.220176865 |
| | | | 5 | -.444666667 | .4681648269 | .361 | -1.46471020 | .5753768647 |

**Figure 10. Screenshot of Tables presenting LSD post-hoc testing of AIL/ASL lignin fractions in Q208 genotype.** The results relate to figure 3d in main document. Screenshots derived from SPSS statistical package. Refer to Table 1 for numbers corresponding to tissue type. Abbreviations, TI: Top Internode; MI: Middle Internode; BI: Bottom Internode; 1$^{st}$ Visible Dewlap Leaf: L1; 5$^{th}$ Visible Dewlap Leaf: L5; R: Root.

**Table 11. Homogenous subsets of AIL/ASL lignin fractions in Q208 genotype as calculated by LSD post-hoc testing.** The results relate to figure 3d in main document. Letters indicate the presence of significant difference between values within the same genotype.

| Tissue Type | Homogenous Subsets | |
|---|---|---|
| | ASL | AIL |
| **BI** | ab | ab |
| **MI** | a | a |
| **TI** | c | b |
| **R** | bc | c |
| **L1** | d | ac |
| **L5** | d | ac |

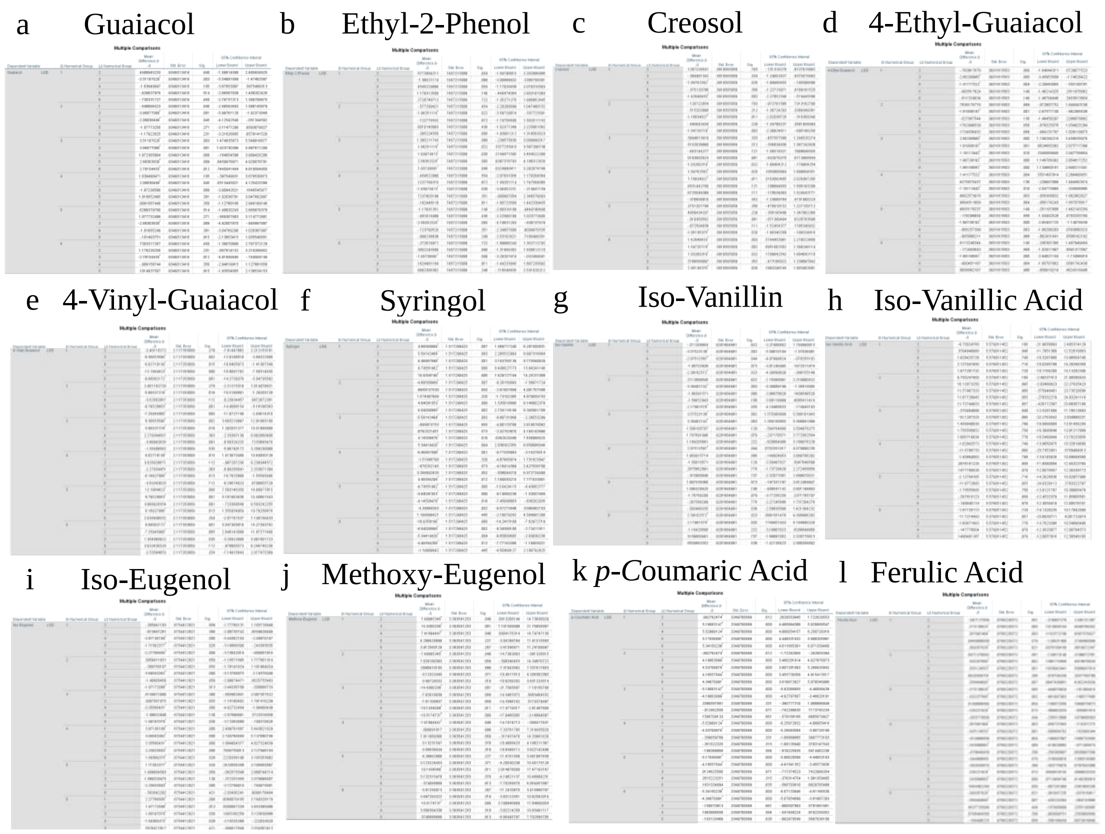


**Figure 11. Screenshot of Tables presenting LSD post-hoc testing of syringyl, guaiacyl and hydroxycinnamyl lignin fractions in KQ228 genotype.** The results relate to figure 4 in main document. Screenshots derived from SPSS statistical package. Refer to Table 1 for numbers corresponding to tissue type. Abbreviations, TI: Top Internode; MI: Middle Internode; BI: Bottom Internode; 1$^{st}$ Visible Dewlap Leaf: L1; 5$^{th}$ Visible Dewlap Leaf: L5; R: Root.

**Table 12. Homogenous subsets of syringyl, guaiacyl and hydroxycinnamyl lignin fractions in KQ228 genotype as calculated by LSD post-hoc testing.** The results relate to figure 4 in main document. Letters indicate the presence of significant difference between values within the same genotype.

| Tissue Type | Homogenous Subsets | | | | | | | | | | | |
|---|---|---|---|---|---|---|---|---|---|---|---|---|
| | Guaiacol | Ethyl-2-Phenol | Creosol | 4-Ethyl-Guaiacol | 4-vinyl-guaiacol | Syringol | Iso-Vanillin | Iso-Vanillic acid | Iso-Eugenol | Methoxy-Eugenol | *p*-coumaric acid | Ferulic Acid |
| **BI** | a | ab | a | a | a | a | a | a | a | a | a | a |
| **MI** | a | b | a | ab | ab | b | a | a | ab | ab | b | a |
| **TI** | b | a | ab | c | cd | b | b | a | ab | b | c | c |
| **R** | ab | b | bc | b | bc | bc | ab | a | d | ab | c | c |
| **L1** | b | b | ab | ab | d | cd | ab | a | bc | a | c | ab |
| **L5** | b | ab | c | ab | cd | d | b | a | c | a | c | bc |

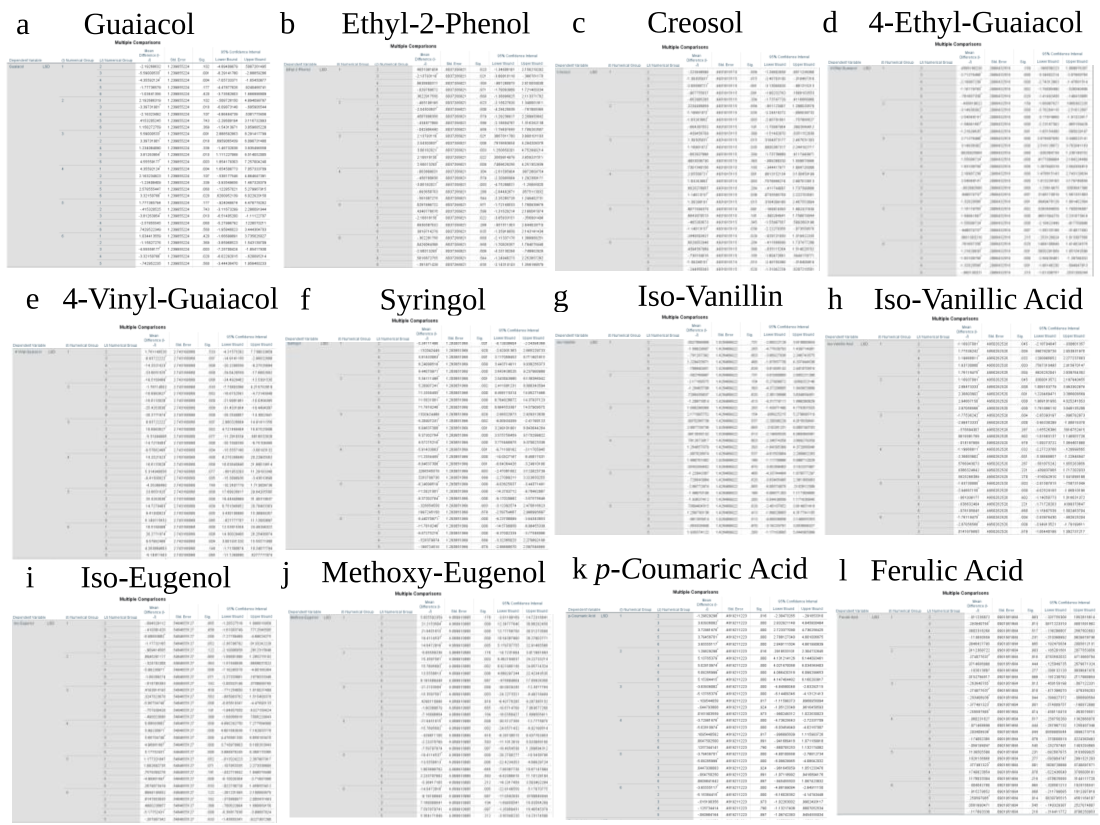


**Figure 12. Screenshot of Tables presenting LSD post-hoc testing of syringyl, guaiacyl and hydroxycinnamyl lignin fractions in Q208 genotype.** The results relate to figure 4 in main document. Screenshots derived from SPSS statistical package. Refer to Table 1 for numbers corresponding to tissue type. Abbreviations, TI: Top Internode; MI: Middle Internode; BI: Bottom Internode; 1$^{st}$ Visible Dewlap Leaf: L1; 5$^{th}$ Visible Dewlap Leaf: L5; R: Root.

**Table 13. Homogenous subsets of syringyl, guaiacyl and hydroxycinnamyl lignin fractions in Q208 genotype as calculated by LSD post-hoc testing.** The results relate to figure 4 in main document. Letters indicate the presence of significant difference between values within the same genotype.

| Tissue Type | Homogenous Subsets | | | | | | | | | | | |
|---|---|---|---|---|---|---|---|---|---|---|---|---|
| | Guaiacol | Ethyl-2-Phenol | Creosol | 4-Ethyl-Guaiacol | 4-vinyl-guaiacol | Syringol | Iso-Vanillin | Iso-Vanillic acid | Iso-Eugenol | Methoxy-Eugenol | p-coumaric acid | Ferulic Acid |
| **BI** | a | a | a | a | a | a | a | a | a | a | a | a |
| **MI** | ab | a | a | a | a | b | a | b | a | ab | b | a |
| **TI** | c | b | bc | b | b | a | a | c | a | c | c | b |
| **R** | bc | a | c | b | bc | c | a | c | b | c | c | a |
| **L1** | ab | a | ab | c | cd | c | a | c | a | c | c | a |
| **L5** | ab | a | ab | c | d | c | a | c | a | bc | c | a |

**Multiple Comparisons**

Dependent Variable: S/G ratio

LSD

| (I) Numerical Group Identifier | (J) Numerical Group Identifier | Mean Difference (I-J) | Std. Error | Sig. | 95% Confidence Interval Lower Bound | 95% Confidence Interval Upper Bound |
|---|---|---|---|---|---|---|
| 1 | 2 | .433521508* | .1079259435 | .002 | .1983710774 | .6686719381 |
| | 3 | .658275918* | .1079259435 | .000 | .4231254878 | .8934263485 |
| | 4 | .491856800* | .1079259435 | .001 | .2567063693 | .7270072300 |
| | 5 | .507922767* | .1079259435 | .001 | .2727723371 | .7430731978 |
| | 6 | .530403740* | .1079259435 | .000 | .2952533094 | .7655541701 |
| 2 | 1 | -.433521508* | .1079259435 | .002 | -.668671938 | -.198371077 |
| | 3 | .2247544104 | .1079259435 | .059 | -.010396020 | .4599048407 |
| | 4 | .0583352919 | .1079259435 | .599 | -.176815138 | .2934857222 |
| | 5 | .0744012597 | .1079259435 | .504 | -.160749171 | .3095516901 |
| | 6 | .0968822320 | .1079259435 | .387 | -.138268198 | .3320326624 |
| 3 | 1 | -.658275918* | .1079259435 | .000 | -.893426348 | -.423125488 |
| | 2 | -.224754410 | .1079259435 | .059 | -.459904841 | .0103960199 |
| | 4 | -.166419119 | .1079259435 | .149 | -.401569549 | .0687313118 |
| | 5 | -.150353151 | .1079259435 | .189 | -.385503581 | .0847972796 |
| | 6 | -.127872178 | .1079259435 | .259 | -.363022609 | .1072782519 |
| 4 | 1 | -.491856800* | .1079259435 | .001 | -.727007230 | -.256706369 |
| | 2 | -.058335292 | .1079259435 | .599 | -.293485722 | .1768151384 |
| | 3 | .1664191185 | .1079259435 | .149 | -.068731312 | .4015695488 |
| | 5 | .0160659678 | .1079259435 | .884 | -.219084463 | .2512163982 |
| | 6 | .0385469401 | .1079259435 | .727 | -.196603490 | .2736973704 |
| 5 | 1 | -.507922767* | .1079259435 | .001 | -.743073198 | -.272772337 |
| | 2 | -.074401260 | .1079259435 | .504 | -.309551690 | .1607491706 |
| | 3 | .1503531507 | .1079259435 | .189 | -.084797280 | .3855035810 |
| | 4 | -.016065968 | .1079259435 | .884 | -.251216398 | .2190844625 |
| | 6 | .0224809723 | .1079259435 | .838 | -.212669458 | .2576314026 |
| 6 | 1 | -.530403740* | .1079259435 | .000 | -.765554170 | -.295253309 |
| | 2 | -.096882232 | .1079259435 | .387 | -.332032662 | .1382681983 |
| | 3 | .1278721784 | .1079259435 | .259 | -.107278252 | .3630226087 |
| | 4 | -.038546940 | .1079259435 | .727 | -.273697370 | .1966034902 |
| | 5 | -.022480972 | .1079259435 | .838 | -.257631403 | .2126694580 |

*. The mean difference is significant at the 0.05 level.

**Figure 13. Screenshot of Table presenting LSD post-hoc testing of syringyl and guaiacyl lignin ratio in KQ228 genotype.** The results relate to table 2 in main document. Screenshots derived from SPSS statistical package. Refer to Table 1 for numbers corresponding to tissue type. Abbreviations, TI: Top Internode; MI: Middle Internode; BI: Bottom Internode; $1^{st}$ Visible Dewlap Leaf: L1; $5^{th}$ Visible Dewlap Leaf: L5; R: Root.

**Table 14. Homogenous subsets of syringyl and guaiacyl lignin in KQ228 genotype as calculated by LSD post-hoc testing.** The results relate to table 2 in main document. Letters indicate the presence of significant difference between values within the same genotype.

| Tissue Type | Homogenous Subsets: Syringyl/Guaiacyl Ratio |
|---|---|
| **BI** | a |
| **MI** | b |
| **TI** | b |
| **R** | b |
| **L1** | b |
| **L5** | b |

**Multiple Comparisons**

Dependent Variable: S/G ratio

LSD

| (I) Numerical Group Identifier | (J) Numerical Group Identifier | Mean Difference (I-J) | Std. Error | Sig. | 95% Confidence Interval | |
|---|---|---|---|---|---|---|
| | | | | | Lower Bound | Upper Bound |
| 7 | 8 | .0325266437 | .0936362316 | .734 | -.171489179 | .2365424663 |
| | 9 | .599875107* | .0936362316 | .000 | .3958592846 | .8038909298 |
| | 10 | .712561652* | .0936362316 | .000 | .5085458293 | .9165774745 |
| | 11 | .688555201* | .0936362316 | .000 | .4845393787 | .8925710239 |
| | 12 | .592722249* | .0936362316 | .000 | .3887064265 | .7967380718 |
| 8 | 7 | -.032526644 | .0936362316 | .734 | -.236542466 | .1714891790 |
| | 9 | .567348464* | .0936362316 | .000 | .3633326409 | .7713642862 |
| | 10 | .680035008* | .0936362316 | .000 | .4760191856 | .8840508309 |
| | 11 | .656028558* | .0936362316 | .000 | .4520127350 | .8600443803 |
| | 12 | .560195605* | .0936362316 | .000 | .3561797828 | .7642114281 |
| 9 | 7 | -.599875107* | .0936362316 | .000 | -.803890930 | -.395859285 |
| | 8 | -.567348464* | .0936362316 | .000 | -.771364286 | -.363332641 |
| | 10 | .1126865447 | .0936362316 | .252 | -.091329278 | .3167023673 |
| | 11 | .0886800941 | .0936362316 | .362 | -.115335729 | .2926959167 |
| | 12 | -.007152858 | .0936362316 | .940 | -.211168681 | .1968629646 |
| 10 | 7 | -.712561652* | .0936362316 | .000 | -.916577475 | -.508545829 |
| | 8 | -.680035008* | .0936362316 | .000 | -.884050831 | -.476019186 |
| | 9 | -.112686545 | .0936362316 | .252 | -.316702367 | .0913292780 |
| | 11 | -.024006451 | .0936362316 | .802 | -.228022273 | .1800093721 |
| | 12 | -.119839403 | .0936362316 | .225 | -.323855225 | .0841764199 |
| 11 | 7 | -.688555201* | .0936362316 | .000 | -.892571024 | -.484539379 |
| | 8 | -.656028558* | .0936362316 | .000 | -.860044380 | -.452012735 |
| | 9 | -.088680094 | .0936362316 | .362 | -.292695917 | .1153357285 |
| | 10 | .0240064506 | .0936362316 | .802 | -.180009372 | .2280222732 |
| | 12 | -.095832952 | .0936362316 | .326 | -.299848775 | .1081828705 |
| 12 | 7 | -.592722249* | .0936362316 | .000 | -.796738072 | -.388706427 |
| | 8 | -.560195605* | .0936362316 | .000 | -.764211428 | -.356179783 |
| | 9 | .0071528581 | .0936362316 | .940 | -.196862965 | .2111686807 |
| | 10 | .1198394028 | .0936362316 | .225 | -.084176420 | .3238552254 |
| | 11 | .0958329522 | .0936362316 | .326 | -.108182870 | .2998487748 |

*. The mean difference is significant at the 0.05 level.

**Figure 14. Screenshot of Table presenting LSD post-hoc testing of syringyl and guaiacyl lignin ratio in Q208 genotype.** The results relate to table 2 in main document. Screenshots derived from SPSS statistical package. Refer to Table 1 for numbers corresponding to tissue type. Abbreviations, TI: Top Internode; MI: Middle Internode; BI: Bottom Internode; 1st Visible Dewlap Leaf: L1; 5th Visible Dewlap Leaf: L5; R: Root.

**Table 15. Homogenous subsets of syringyl and guaiacyl lignin in Q208 genotype as calculated by LSD post-hoc testing.** The results relate to table 2 in main document. Letters indicate the presence of significant difference between values within the same genotype.

| Tissue Type | Homogenous Subsets |
|---|---|
| | Syringyl/Guaiacyl Ratio |
| **BI** | a |
| **MI** | a |
| **TI** | b |
| **R** | b |
| **L1** | b |
| **L5** | b |

**Multiple Comparisons**

| Dependent Variable | | (I) Numerical Group | (J) Numerical Group | Mean Difference (I-J) | Std. Error | Sig. | 95% Confidence Interval | |
|---|---|---|---|---|---|---|---|---|
| | | | | | | | Lower Bound | Upper Bound |
| Glucose Release % | LSD | 1 | 2 | -22.2943647* | 9.306805957 | .034 | -42.5721530 | -2.01657653 |
| | | | 3 | -34.3435898* | 9.306805957 | .003 | -54.6213780 | -14.0658015 |
| | | | 4 | .9705516521 | 9.306805957 | .919 | -19.3072366 | 21.24833987 |
| | | | 5 | 7.853397486 | 9.306805957 | .415 | -12.4243907 | 28.13118571 |
| | | | 6 | 14.91371725 | 9.306805957 | .135 | -5.36407098 | 35.19150547 |
| | | 2 | 1 | 22.2943647* | 9.306805957 | .034 | 2.016576527 | 42.57215297 |
| | | | 3 | -12.0492250 | 9.306805957 | .220 | -32.3270132 | 8.228563205 |
| | | | 4 | 23.2649164* | 9.306805957 | .028 | 2.987128179 | 43.54270462 |
| | | | 5 | 30.1477622* | 9.306805957 | .007 | 9.869974013 | 50.42555046 |
| | | | 6 | 37.2080820* | 9.306805957 | .002 | 16.93029377 | 57.48587022 |
| | | 3 | 1 | 34.3435898* | 9.306805957 | .003 | 14.06580154 | 54.62137799 |
| | | | 2 | 12.04922502 | 9.306805957 | .220 | -8.22856321 | 32.32701324 |
| | | | 4 | 35.3141414* | 9.306805957 | .003 | 15.03635320 | 55.59192964 |
| | | | 5 | 42.1969873* | 9.306805957 | .001 | 21.91919903 | 62.47477547 |
| | | | 6 | 49.2573070* | 9.306805957 | .000 | 28.97951879 | 69.53509523 |
| | | 4 | 1 | -.970551652 | 9.306805957 | .919 | -21.2483399 | 19.30723657 |
| | | | 2 | -23.2649164* | 9.306805957 | .028 | -43.5427046 | -2.98712818 |
| | | | 3 | -35.3141414* | 9.306805957 | .003 | -55.5919296 | -15.0363532 |
| | | | 5 | 6.882845834 | 9.306805957 | .474 | -13.3949424 | 27.16063406 |
| | | | 6 | 13.94316559 | 9.306805957 | .160 | -6.33462263 | 34.22095382 |
| | | 5 | 1 | -7.85339749 | 9.306805957 | .415 | -28.1311857 | 12.42439074 |
| | | | 2 | -30.1477622* | 9.306805957 | .007 | -50.4255505 | -9.86997401 |
| | | | 3 | -42.1969873* | 9.306805957 | .001 | -62.4747755 | -21.9191990 |
| | | | 4 | -6.88284583 | 9.306805957 | .474 | -27.1606341 | 13.39494239 |
| | | | 6 | 7.060319761 | 9.306805957 | .463 | -13.2174685 | 27.33810798 |
| | | 6 | 1 | -14.9137172 | 9.306805957 | .135 | -35.1915055 | 5.364070976 |
| | | | 2 | -37.2080820* | 9.306805957 | .002 | -57.4858702 | -16.9302938 |
| | | | 3 | -49.2573070* | 9.306805957 | .000 | -69.5350952 | -28.9795188 |
| | | | 4 | -13.9431656 | 9.306805957 | .160 | -34.2209538 | 6.334622628 |
| | | | 5 | -7.06031976 | 9.306805957 | .463 | -27.3381080 | 13.21746846 |
| Xylose Release % | LSD | 1 | 2 | -30.9280264* | 3.361686012 | .000 | -38.2525110 | -23.6035418 |
| | | | 3 | -29.6253123* | 3.361686012 | .000 | -36.9497969 | -22.3008277 |
| | | | 4 | 3.364702266 | 3.361686012 | .337 | -3.95978235 | 10.68918688 |
| | | | 5 | 11.8712168* | 3.361686012 | .004 | 4.546732172 | 19.19570140 |
| | | | 6 | 12.5977014* | 3.361686012 | .003 | 5.273216738 | 19.92218596 |
| | | 2 | 1 | 30.9280264* | 3.361686012 | .000 | 23.60354181 | 38.25251104 |
| | | | 3 | 1.302714158 | 3.361686012 | .705 | -6.02177046 | 8.627198771 |
| | | | 4 | 34.2927287* | 3.361686012 | .000 | 26.96824408 | 41.61721330 |
| | | | 5 | 42.7992432* | 3.361686012 | .000 | 35.47475860 | 50.12372782 |
| | | | 6 | 43.5257278* | 3.361686012 | .000 | 36.20124316 | 50.85021239 |
| | | 3 | 1 | 29.6253123* | 3.361686012 | .000 | 22.30082765 | 36.94979688 |
| | | | 2 | -1.30271416 | 3.361686012 | .705 | -8.62719877 | 6.021770455 |
| | | | 4 | 32.9900145* | 3.361686012 | .000 | 25.66552992 | 40.31449915 |
| | | | 5 | 41.4965291* | 3.361686012 | .000 | 34.17204444 | 48.82101366 |
| | | | 6 | 42.2230136* | 3.361686012 | .000 | 34.89852901 | 49.54749823 |
| | | 4 | 1 | -3.36470227 | 3.361686012 | .337 | -10.6891869 | 3.959782347 |
| | | | 2 | -34.2927287* | 3.361686012 | .000 | -41.6172133 | -26.9682441 |
| | | | 3 | -32.9900145* | 3.361686012 | .000 | -40.3144991 | -25.6655299 |
| | | | 5 | 8.50651452* | 3.361686012 | .026 | 1.182029905 | 15.83099913 |
| | | | 6 | 9.23299909* | 3.361686012 | .018 | 1.908514472 | 16.55748370 |
| | | 5 | 1 | -11.8712168* | 3.361686012 | .004 | -19.1957014 | -4.54673217 |
| | | | 2 | -42.7992432* | 3.361686012 | .000 | -50.1237278 | -35.4747586 |
| | | | 3 | -41.4965291* | 3.361686012 | .000 | -48.8210137 | -34.1720444 |
| | | | 4 | -8.50651452* | 3.361686012 | .026 | -15.8309991 | -1.18202991 |
| | | | 6 | .7264845667 | 3.361686012 | .833 | -6.59800005 | 8.050969180 |
| | | 6 | 1 | -12.5977014* | 3.361686012 | .003 | -19.9221860 | -5.27321674 |
| | | | 2 | -43.5257278* | 3.361686012 | .000 | -50.8502124 | -36.2012432 |
| | | | 3 | -42.2230136* | 3.361686012 | .000 | -49.5474982 | -34.8985290 |
| | | | 4 | -9.23299909* | 3.361686012 | .018 | -16.5574837 | -1.90851447 |
| | | | 5 | -.726484567 | 3.361686012 | .833 | -8.05096918 | 6.598000047 |

*. The mean difference is significant at the 0.05 level.

**Figure 15. Screenshot of Table presenting LSD post-hoc testing of xylan and glucan release in the KQ228 genotype.** The results relate to figure 5 in main document. Screenshots derived from SPSS statistical package. Refer to Table 1 for numbers corresponding to tissue type. Abbreviations, TI: Top Internode; MI: Middle Internode; BI: Bottom Internode; $1^{st}$ Visible Dewlap Leaf: L1; $5^{th}$ Visible Dewlap Leaf: L5; R: Root.

**Table 16. Homogenous subsets of xylan and glucan release in Q208 genotype as calculated by LSD post-hoc testing.** The results relate to figure 5 in main document. Letters indicate the presence of significant difference between values within the same genotype.

| Tissue Type | Homogenous Subsets | |
|---|---|---|
| | Glucan Release % | Xylan Release % |
| **BI** | bc | b |
| **MI** | ab | a |
| **TI** | a | a |
| **R** | c | b |
| **L1** | c | c |
| **L5** | c | c |

**Multiple Comparisons**

| Dependent Variable | | (I) Numerical Group | (J) Numerical Group | Mean Difference (I-J) | Std. Error | Sig. | 95% Confidence Interval Lower Bound | 95% Confidence Interval Upper Bound |
|---|---|---|---|---|---|---|---|---|
| Glucose Release % | LSD | 7 | 8 | 14.5827444* | 6.107806446 | .034 | 1.274977364 | 27.89051146 |
| | | | 9 | -15.5075696* | 6.107806446 | .026 | -28.8153367 | -2.19980258 |
| | | | 10 | 24.6174198* | 6.107806446 | .002 | 11.30965277 | 37.92518686 |
| | | | 11 | 11.42515747 | 6.107806446 | .086 | -1.88260957 | 24.73292452 |
| | | | 12 | 17.6250305* | 6.107806446 | .014 | 4.317263444 | 30.93279753 |
| | | 8 | 7 | -14.5827444* | 6.107806446 | .034 | -27.8905115 | -1.27497736 |
| | | | 9 | -30.0903140* | 6.107806446 | .000 | -43.3980811 | -16.7825470 |
| | | | 10 | 10.03467541 | 6.107806446 | .126 | -3.27309164 | 23.34244245 |
| | | | 11 | -3.15758694 | 6.107806446 | .615 | -16.4653540 | 10.15018011 |
| | | | 12 | 3.042286080 | 6.107806446 | .627 | -10.2654810 | 16.35005313 |
| | | 9 | 7 | 15.5075696* | 6.107806446 | .026 | 2.199802579 | 28.81533667 |
| | | | 8 | 30.0903140* | 6.107806446 | .000 | 16.78254699 | 43.39808108 |
| | | | 10 | 40.1249894* | 6.107806446 | .000 | 26.81722239 | 53.43275649 |
| | | | 11 | 26.9327271* | 6.107806446 | .001 | 13.62496005 | 40.24049414 |
| | | | 12 | 33.1326001* | 6.107806446 | .000 | 19.82483307 | 46.44036716 |
| | | 10 | 7 | -24.6174198* | 6.107806446 | .002 | -37.9251869 | -11.3096528 |
| | | | 8 | -10.0346754 | 6.107806446 | .126 | -23.3424425 | 3.273091639 |
| | | | 9 | -40.1249894* | 6.107806446 | .000 | -53.4327565 | -26.8172224 |
| | | | 11 | -13.1922623 | 6.107806446 | .052 | -26.5000294 | .1155047025 |
| | | | 12 | -6.99238933 | 6.107806446 | .275 | -20.3001564 | 6.315377719 |
| | | 11 | 7 | -11.4251575 | 6.107806446 | .086 | -24.7329245 | 1.882609572 |
| | | | 8 | 3.157586936 | 6.107806446 | .615 | -10.1501801 | 16.46535398 |
| | | | 9 | -26.9327271* | 6.107806446 | .001 | -40.2404941 | -13.6249601 |
| | | | 10 | 13.19226234 | 6.107806446 | .052 | -.115504703 | 26.50002939 |
| | | | 12 | 6.199873016 | 6.107806446 | .330 | -7.10789403 | 19.50764006 |
| | | 12 | 7 | -17.6250305* | 6.107806446 | .014 | -30.9327975 | -4.31726344 |
| | | | 8 | -3.04228608 | 6.107806446 | .627 | -16.3500531 | 10.26548097 |
| | | | 9 | -33.1326001* | 6.107806446 | .000 | -46.4403672 | -19.8248331 |
| | | | 10 | 6.992389327 | 6.107806446 | .275 | -6.31537772 | 20.30015637 |
| | | | 11 | -6.19987302 | 6.107806446 | .330 | -19.5076401 | 7.107894029 |
| Xylose Release % | LSD | 7 | 8 | 7.51675457* | 2.284500701 | .006 | 2.539255130 | 12.49425401 |
| | | | 9 | -14.1120239* | 2.284500701 | .000 | -19.0895233 | -9.13452445 |
| | | | 10 | 10.7821379* | 2.284500701 | .000 | 5.804638492 | 15.75963737 |
| | | | 11 | 15.1029708* | 2.284500701 | .000 | 10.12547135 | 20.08047023 |
| | | | 12 | 16.3424267* | 2.284500701 | .000 | 11.36492727 | 21.31992614 |
| | | 8 | 7 | -7.51675457* | 2.284500701 | .006 | -12.4942540 | -2.53925513 |
| | | | 9 | -21.6287785* | 2.284500701 | .000 | -26.6062779 | -16.6512790 |
| | | | 10 | 3.265383362 | 2.284500701 | .178 | -1.71211608 | 8.242882800 |
| | | | 11 | 7.58621622* | 2.284500701 | .006 | 2.608716785 | 12.56371566 |
| | | | 12 | 8.82567214* | 2.284500701 | .002 | 3.848172698 | 13.80317157 |
| | | 9 | 7 | 14.1120239* | 2.284500701 | .000 | 9.134524452 | 19.08952333 |
| | | | 8 | 21.6287785* | 2.284500701 | .000 | 16.65127902 | 26.60627789 |
| | | | 10 | 24.8941618* | 2.284500701 | .000 | 19.91666238 | 29.87166126 |
| | | | 11 | 29.2149947* | 2.284500701 | .000 | 24.23749524 | 34.19249412 |
| | | | 12 | 30.4544506* | 2.284500701 | .000 | 25.47695116 | 35.43195003 |
| | | 10 | 7 | -10.7821379* | 2.284500701 | .000 | -15.7596374 | -5.80463849 |
| | | | 8 | -3.26538336 | 2.284500701 | .178 | -8.24288280 | 1.712116075 |
| | | | 9 | -24.8941618* | 2.284500701 | .000 | -29.8716613 | -19.9166624 |
| | | | 11 | 4.320832861 | 2.284500701 | .083 | -.656666577 | 9.298332298 |
| | | | 12 | 5.56028877* | 2.284500701 | .032 | .5827893359 | 10.53778821 |
| | | 11 | 7 | -15.1029708* | 2.284500701 | .000 | -20.0804702 | -10.1254714 |
| | | | 8 | -7.58621622* | 2.284500701 | .006 | -12.5637157 | -2.60871679 |
| | | | 9 | -29.2149947* | 2.284500701 | .000 | -34.1924941 | -24.2374952 |
| | | | 10 | -4.32083286 | 2.284500701 | .083 | -9.29833230 | .6566665769 |
| | | | 12 | 1.239455913 | 2.284500701 | .597 | -3.73804352 | 6.216955350 |
| | | 12 | 7 | -16.3424267* | 2.284500701 | .000 | -21.3199261 | -11.3649273 |
| | | | 8 | -8.82567214* | 2.284500701 | .002 | -13.8031716 | -3.84817270 |
| | | | 9 | -30.4544506* | 2.284500701 | .000 | -35.4319500 | -25.4769512 |
| | | | 10 | -5.56028877* | 2.284500701 | .032 | -10.5377882 | -.582789336 |
| | | | 11 | -1.23945591 | 2.284500701 | .597 | -6.21695535 | 3.738043525 |

*. The mean difference is significant at the 0.05 level.

**Figure 16. Screenshot of Table presenting LSD post-hoc testing of xylan and glucan release in Q208 genotype.** The results relate to figure 5 in main document. Screenshots derived from SPSS statistical package. Refer to Table 1 for numbers corresponding to tissue type. Abbreviations, TI: Top Internode; MI: Middle Internode; BI: Bottom Internode; 1st Visible Dewlap Leaf: L1; 5th Visible Dewlap Leaf: L5; R: Root.

**Table 16. Homogenous subsets of xylan and glucan release in Q208 genotype as calculated by LSD post-hoc testing.** The results relate to figure 5 in main document. Letters indicate the presence of significant difference between values within the same genotype.

| Tissue Type | Homogenous Subsets | |
|---|---|---|
| | Glucan Release % | Xylan Release % |
| **BI** | b | b |
| **MI** | bc | c |
| **TI** | a | a |
| **R** | c | cd |
| **L1** | bc | cd |
| **L5** | c | cd |
